# In-Flow Heterogeneous Triplet–Triplet Annihilation Upconversion

**DOI:** 10.1021/acsphyschemau.3c00062

**Published:** 2024-03-12

**Authors:** Jorge Castellanos-Soriano, Francisco Garnes-Portolés, M. Consuelo Jiménez, Antonio Leyva-Pérez, Raúl Pérez-Ruiz

**Affiliations:** † Departamento de Química, Universitat Politècnica de València (UPV), Camino de Vera, S/N 46022 Valencia, Spain; ‡ Instituto de Tecnología Química (ITQ), Universitat Politècnica de València-Consejo Superior de Investigaciones Científicas (UPV-CSIC), Av. de los Naranjos, S/N 46022 Valencia, Spain

**Keywords:** Heterogeneous, TTA, continuous-flow, visible light, Mizoroki−Heck

## Abstract

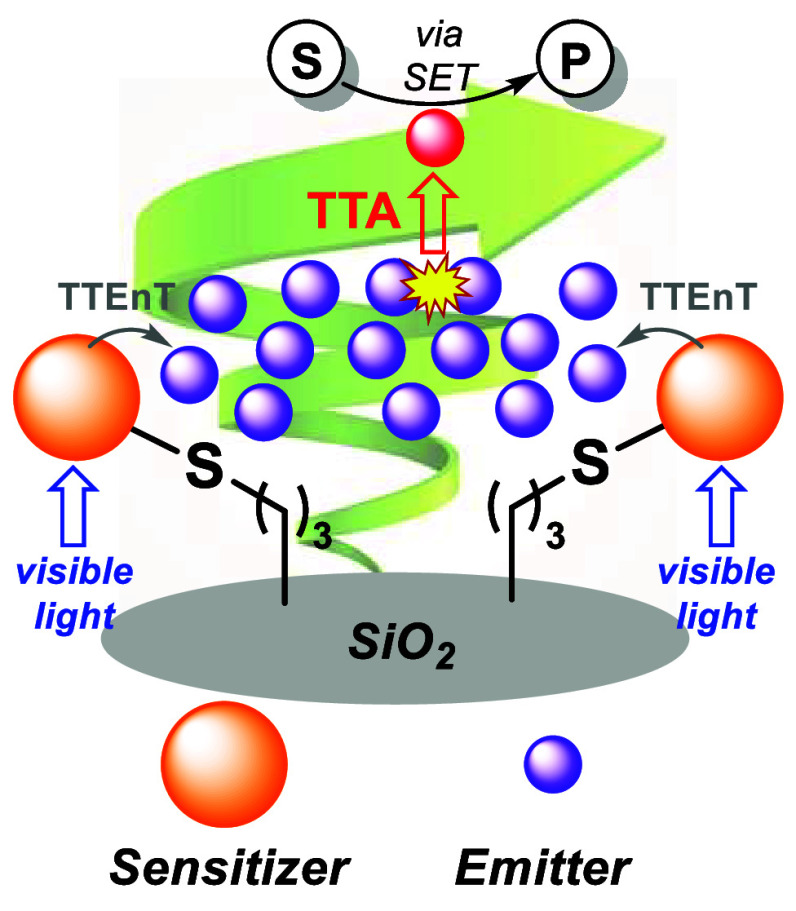

Photon upconversion based on triplet–triplet annihilation (TTA-UC) is an attractive wavelength conversion with increasing use in organic synthesis in the homogeneous phase; however, this technology has not performed with canonical solid catalysts yet. Herein, a BOPHY dye covalently anchored on silica is successfully used as a sensitizer in a TTA system that efficiently catalyzes Mizoroki–Heck coupling reactions. This procedure has enabled the implementation of in-flow reaction conditions for the synthesis of a variety of aromatic compounds, and mechanistic proof has been obtained by means of transient absorption spectroscopy.

The interest for multiphoton photoredox catalysis^1−6^ has experienced a considerable growth in the last years, being a powerful tool to circumvent the thermodynamic and redox limitations of conventional photoredox catalysts.^7,8^ In particular, the biphotonic process based on triplet–triplet annihilation (TTA) has been given substantial attention since this phenomenon is currenlty one of the most attractive wavelength conversion procedures.^9−11^ It comprises a bimolecular system (sensitizer or donor + annihilator or acceptor); even though the annihilator is not directly excited, formation of its lowest triplet excited state is achieved through triplet–triplet energy transfer (TTEnT) from the primary sensitizer. Subsequently, the TTA event produces the fluorescent singlet excited state of the annihilator, which efficiently emits higher energy than the one employed at excitation (lower). Indeed, this singlet state can get involved in electron/energy transfer processes, allowing the activation of substrates for organic synthetic purposes.^3,12,13^ To note, a higher energetic source such as UV light is typically required to generate the annihilator singlet state, making the TTA approach more attractive and advantageous since much lower-energy input radiation can be employed.

On the other hand, flow chemistry technology is considered an important tool to overcome some typical limitations of batch synthesis such as slow heat and mass transfer, offering the possibility to shorten reaction times and, in some cases, to increase selectivities as well as to enable scale-up; in other words, to enable process intensification in a tightly controlled environment. Based on these advantages, continuous-flow photocatalysis represents an important milestone in the pathway of developing milder and more efficient synthetic processes to create new C–C and/or C–X bonds (X = O, N, S,...).^14^ In this way, continuous-flow photocatalysis has been successfully applied in the synthesis of organic pharmaceuticals,^15^ photodegradation of organic compounds,^16^ and photoredox catalysis.^17^

Despite the fact that different TTA upconversion systems have been developed, it appears surprising that exclusively homogeneous phases in batch conditions have been investigated so far, whereas application of in-flow or heterogeneous TTA upconversion to photochemical transformations has not been targeted yet. We have recently performed a coupling reaction using a photocatalytic TTA system under flow settings as a preliminary result.^18^ In this study, although desired products were successfully obtained, yields did not improve as much as those for the batch reaction, presumably due to decomposition of the sensitizer (a BOPHY dye).

A possible strategy to solve this issue might be the immobilization of the sensitizer not only to evade its degradation but also to achieve their successful recovery and reuse.^19^ In this context, silica-based materials are typical support entities because of their facile surface functionalization, low cost, and inertness.^20^ The construction of a silica shell covalently functionalized with a BOPHY dye could thus provide suitable conditions to accomplish chemical transformations via in-flow heterogeneous TTA upconversion. As a proof of concept, a C–C coupling reaction photocatalyzed by a heterogeneous TTA system under continuous-flow setting conditions is herein reported for the first time, as far as we know (Figure 1, a very recent review confirms the novelty of this approach).^21^ Since the TTA process relies on molecular collisions between the triplet acceptors, high mobility of the acceptor is desired. Therefore, covalent bonds are used to immobilize the BOPHY dye (sensitizer) in silica, rather than the 9,10-diphenylanthracene DPA (acceptor).

**Figure 1:**
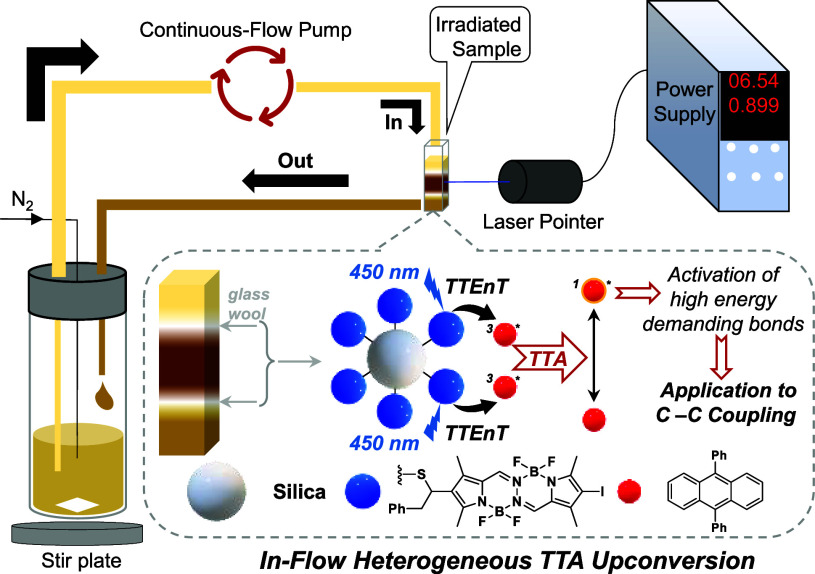
Application of heterogeneous TTA–UC to C–C coupling reactions under continuous-flow conditions.

Hybrid silica@BOPHY material was synthesized according to the following procedure (Scheme 1). We first performed the synthesis of the diiodoBOPHY-like derivative BOPHY-1 by following a previously reported procedure, resulting in an orange solid.^22^ Then, we proceeded with the synthesis of iodoBOPHY-like derivative BOPHY-2 with a reactive alkene group. To this respect, substitution of one iodine atom by a styrene moiety was accomplished through a metal-catalyzed reaction, yielding 84% of the desired product. Finally, BOPHY-2 was susceptible to bond with 3-mercaptopropyl-functionalized silica gel to form the corresponding hybrid material silica@BOPHY-2. Elemental analysis revealed the percentage of organic matter anchored to the solid, which was found to be 5.3 wt % of the solid photocatalyst. The molecular structure of silica@BOPHY-2 was characterized by Fourier-transform infrared spectroscopy (FTIR), magic angle spinning solid nuclear magnetic resonance (MAS-SS-NMR), and steady-state absorption (see details in the Supporting Information (SI)). From the UV–vis spectra (Figure 2), it was clear that the absorption band in the visible region with a maximum at 460 nm for silica@BOPHY-2 in the solid phase matched perfectly with that observed for BOPHY-2 in solution.

**Scheme 1:**
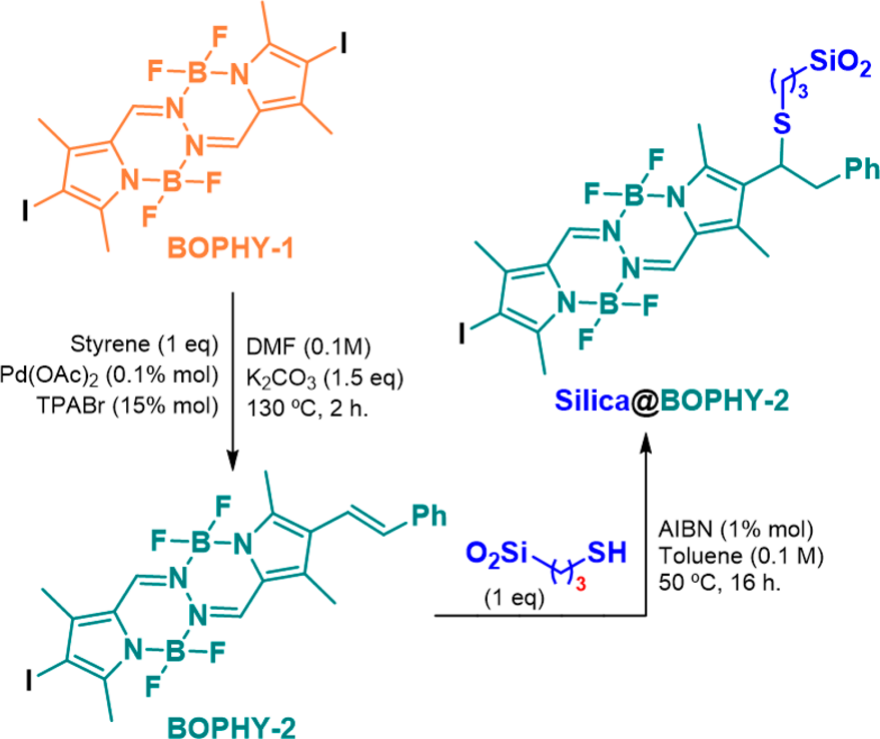
Synthesis of Silica@BOPHY-2

**Figure 2:**
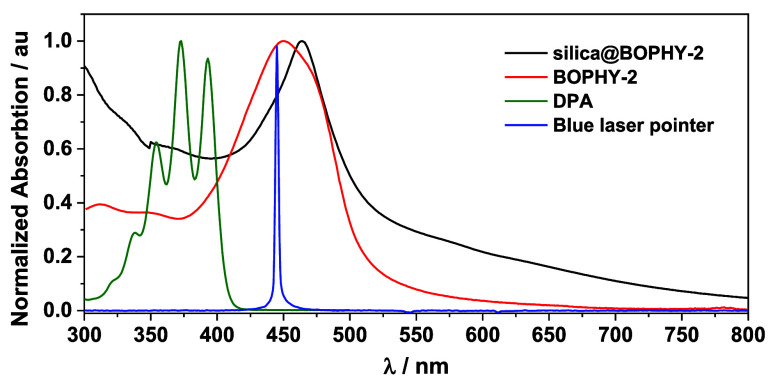
Absorption spectra of silica@BOPHY-2 in the solid phase and both BOPHY-2 (0.01 mM) and DPA (0.01 mM) in aerated ACN/DMA solution. Emission of the laser pointer in blue.

Once the BOPHY dye was immobilized on silica, the next step was to check whether our hybrid silica@BOPHY-2 material could be part of a TTA system for photocatalytic purposes under in-flow heterogeneous conditions. Based on previous successful results on photocatalyzed Mizoroki–Heck reaction for triarylethylenes fabrication using TTA–UC technology,^23^ we decided to afford these challenged C–C couplings by in-flow heterogeneous TTA upconversion (Scheme 2). Here, we placed an anaerobic solution of 4-bromobenzaldehyde, 1,1-diphenylethylene and DPA in a glass bottle. It was delivered to a Pyrex glass holder containing the hybrid silica@BOPHY-2 material by a Fisher continuous-flow pump at 100 rpm through Tygon tubing (ID = 1.6 mm). A blue laser pointer (λ_exc_ = 445 ± 10) was directed to the hybrid material. The final leaving stream was collected again in the glass bottle to continuously evolve the photoreaction (setup photograph in the SI).

**Scheme 2:**
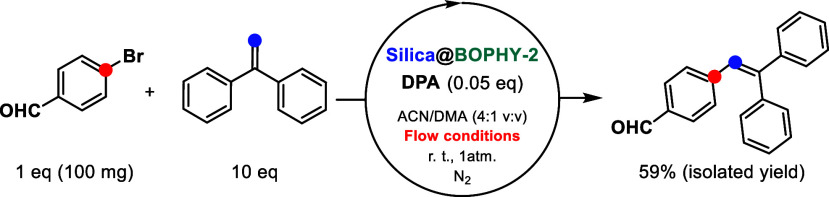
In-Flow Heterogeneous Photocatalytic Mizoroki–Heck Reaction

Optimal conditions implied low reagent loadings and visible light irradiation with the low-cost laser pointer (Table S1 in the SI). The result with the hybrid material was very satisfactory, yielding 60% of the isolated product together with a reaction selectivity of 100%. Control experiments clearly demonstrated that the presence of DPA was essential for this photochemical procedure (see Table S1, entries 5–7 in the SI). It is important to note that the same reaction in the homogeneous phase under similar conditions rather than that performed in the heterogeneous phase (but in this case using 1.2% mol of BOPHY-2 in solution, see procedure A in the SI), either in batch or flow conditions, did result in 21% and 19%, respectively, of the desired product, validating the proposed methodology as a process intensification. More importantly, the reusability of silica@BOPHY-2 retained the photocatalytic activity after 3 cycles tested (see Table S1, entry 3 in the SI). The weak reduction of the silica@BOPHY-2 activity could be explained in terms of adsorption where some amount of the starting material would be adsorbed onto the heterogeneous catalyst in the first run, somehow affecting the next cycles.^24^

These results showcased a metal-free, in-flow catalytic system for the Mizoroki–Heck reaction, which is not easy to find in the literature.^25,26^ Then, we tried to couple aryl chlorides, much more difficult to engage in the Mizoroki–Heck reactions than bromides and iodides, even for palladium catalysts.^27,28^ Indeed, thiophene chloride derivatives coupled well under these metal-free, heterogeneous photocatalytic reaction conditions, with several diphenylethylenes as starting materials (Scheme 3). In all cases, the selectivity of the process was found to be 100% since observation of the reduced product was negligible.

**Scheme 3:**
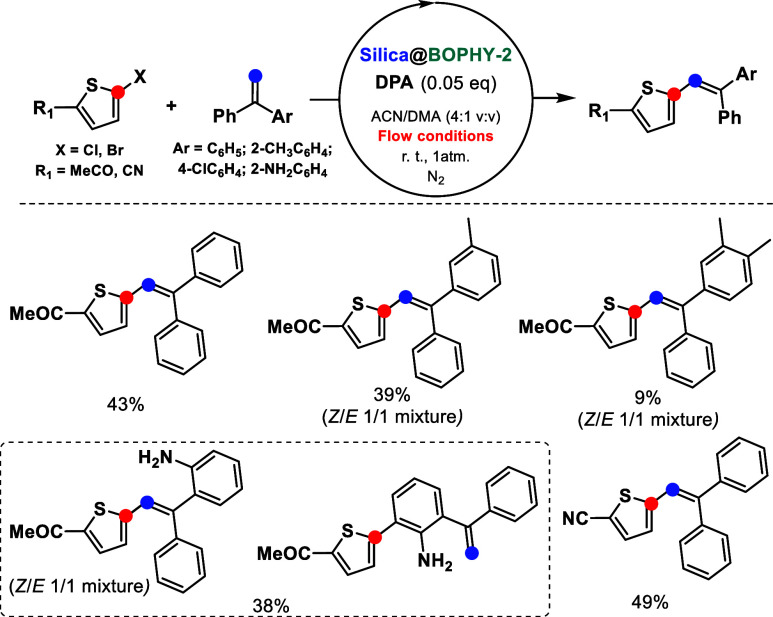
In-Flow Heterogeneous Photocatalytic C–C Couplings

To shed light onto the mechanistic aspects of the above-mentioned process, which involved a photoinduced electron transfer process, transient absorption spectroscopy (TAS) was carried out. Dye BOPHY-2 was chosen as a suitable sensitizer in the bimolecular TTA system, since its structure was analogous to that used in the in-flow heterogeneous conditions whereas DPA was utilized as an emitter. At the first stage, a solution of BOPHY-2 in deaerated *N*,*N*-dimethylacetamide (DMA) and acetonitrile (ACN) mixture was selectively excited (λ_exc_ = 450 nm) by TAS in the μs domain. The T-T absorption band of BOPHY-2 (^3^BOPHY-2*) was observed at 700–750 nm (Figure S3 in the SI), in agreement with literature data,^29^ with a triplet lifetime (τ_T_) determined as 14 μs that fit perfectly to a monoexponential curve (Figure 3A, black line). A gradual decrease of the ^3^BOPHY-2* triplet lifetime was observed in the presence of increasing amounts of DPA (Figure S6). Stern–Volmer analysis (Figure 3B) revealed a quenching rate constant of 4.6 × 10^9^ M^–1^ s^–1^, indicating an efficient triplet–triplet energy transfer (TTEnT) process. To detect the resultant formation of the DPA delayed fluorescence (^1^DPA*) in our conditions, a deaerated DMA/ACN solution of a mixture of BOPHY-2/DPA was submitted to TAS with an excitation of 450 nm. Thus, the upconverted ^1^DPA* was observed displaying the typical emission band (Figure 3C, black line). These results agreed with previously reported data for similar systems.^23^

**Figure 3:**
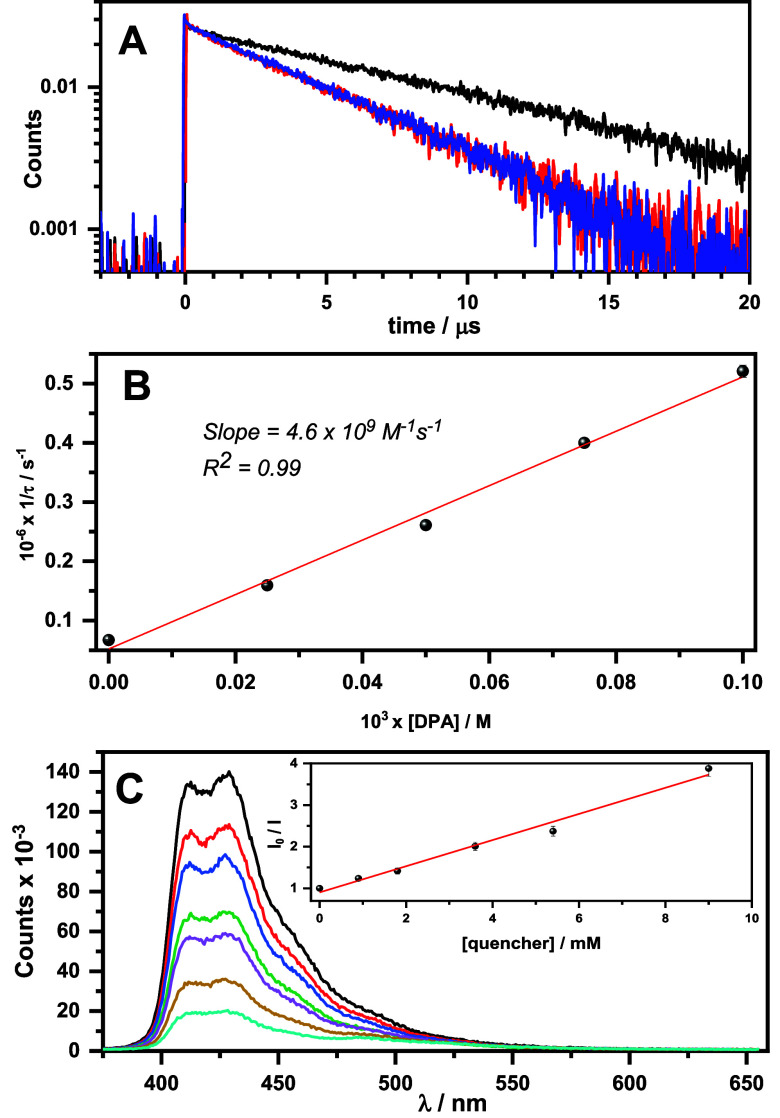
(A) Decays were monitored at 700 nm after 450 nm laser excitation of BOPHY-2 (0.01 mM) in anaerobic ACN/DMA solution without (black) or with 0.1 mM DPA (blue) or with 0.1 mM DPA plus 12 mM 4-bromobenzaldehyde (red). (B) Stern–Volmer analysis was used for the calculation of the corresponding quenching rate constant. (C) Emission spectra (λ_exc_ = 450 nm) of BOPHY-2 (0.01 mM) and DPA (0.1 mM) mixture in anaerobic ACN/DMA solution recorded at 2 μs after the laser pulse, in the presence of increasing amounts of 4-bromobenzaldehyde. Inset: Stern–Volmer plot to obtain *k*_q_ (S_1_); experimental errors were lower than 5% of the obtained results.

Quenching studies demonstrated the interaction between the high-energy delayed fluorescence ^1^DPA* and the aryl bromides through a single electron transfer (SET) process (Figure 3C). A gradual reduction of ^1^DPA* was clearly observed in the presence of increasing amounts of 4-bromobenzaldehyde. By the Stern–Volmer correlation, where *K*_SV_ was estimated as 314 M^–1^ (Figure 3C, inset) and the DPA singlet lifetime value was τ_F_ = 6.96 ns,^8^ the quenching rate constant (*k*_q_) was found to be 4.5 × 10^10^ M^–1^ s^–1^, indicating that SET occurred at a diffusion-controlled rate. Besides, the triplet ^3^BOPHY-2* lifetime in the BOPHY-2/DPA system was not affected by the presence of the corresponding quencher (Figure 3A, blue and red lines) which supported the fact that dye BOPHY-2 was not acting as an activator of the reaction, discarding any SET from the excited BOPHY-2.

We propose a plausible mechanism that is outlined in Scheme 4. Regarding the typical mechanism of TTA-UC, BOPHY-2 is first photoexcited to the excited singlet state (^1^BOPHY-2*), followed by intersystem crossing (ISC) to the excited triplet state (^2^BOPHY-2*). A rapid triplet–triplet energy transfer (TTEnT) occurs to quantitatively produce ^3^DPA*. Triplet–triplet annihilation (TTA) between two ^3^DPA* generates the ^1^DPA* upconverted fluorescence. Now, this highly energetic species ^1^DPA* activates the substrate by SET, leading to the radical ion pair, Ar–Br^–•^ and DPA^+•^.^30^ Fast scission of Ar–Br^–•^ provides the formation of the aryl radical (Ar·) which is successfully trapped by the corresponding nucleophile Nu, giving rise to the radical intermediate *Int-a*. To restore DPA (see Figure S4), SET from *Int-a* to DPA^+•^ occurs and the cationic intermediate *Int-a* is formed which evolves to the final product after deprotonation.

**Scheme 4:**
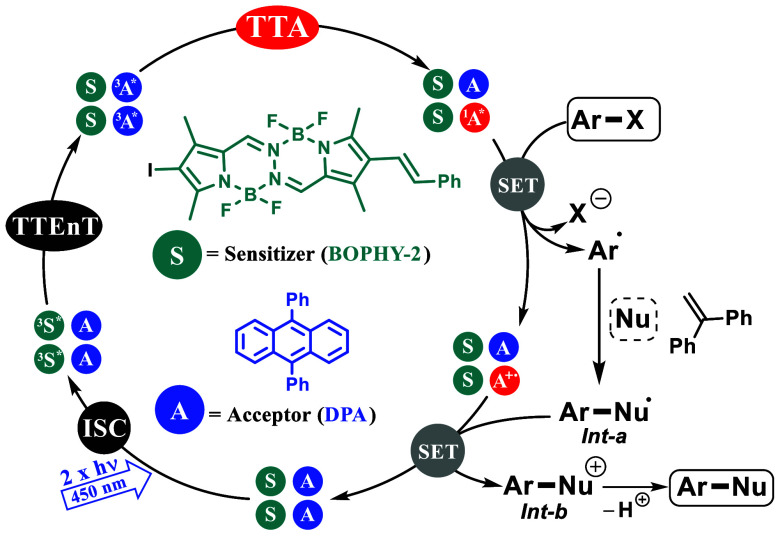
Proposed Reaction Mechanism

Summarizing, we have developed a novel procedure based on a heterogeneous TTA system as a photocatalyst for the construction of new C–C bonds under flow conditions. A BOPHY dye was immobilized onto a silica support (silica@BOPHY) that acted as a sensitizer in the bimolecular TTA system. Then, a continuous-flow solution containing the other partner of the TTA system, and the corresponding reactants, was delivered through the hybrid silica@BOPHY material, which was submitted to visible light irradiation. Product analysis revealed the formation of the desired products. Mechanistic studies by TAS indicated that the most plausible mechanism involved a SET process from the TTA system to the aryl halides. These results open the way to the design of a new photocatalytic process based on heterogeneous TTA systems.

## Supporting Information Available

Materials, general as well as synthetic methods; additional experiments including transient absorption spectroscopy and NMR spectra for all compounds (PDF)
