# Supplementary material for: In-Flow Heterogeneous Triplet–Triplet Annihilation Upconversion
Source: ACS Phys Chem Au. 2024 Mar 12;4(3):242–6. doi: 10.1021/acsphyschemau.3c00062 (PMC11117689; doi:10.1021/acsphyschemau.3c00062)

Supporting Information

In-Flow Heterogeneous Triplet-Triplet Annihilation Upconversion

Jorge Castellanos-Soriano,[a] Francisco Garnes-Portolés,[b] M. Consuelo Jiménez,[a] Antonio Leyva-Pérez,*[b] and Raúl Pérez-Ruiz*[a]

[a] Departamento de Química, Universitat Politècnica de Valencia, Camino de Vera S/N, 46022, Valencia, Spain

[b] Instituto de Tecnología Química (ITQ), Universitat Politècnica de València-Consejo Superior de Investigaciones Científicas (UPV-CSIC), Av. de los Naranjos, S/N 46022 Valencia, Spain.

Table of Contents

This page …… **S1**

1- Materials and methods …… **S2**

2- Synthetic methods …… **S2**

3- Irradiation light source …… **S3**

4- Transient absorption spectroscopy …… **S3**

5- Table S1 …… **S4**

6- Continuous-flow set-up (Figures S1 & S2) …… **S5**

7- Figure S3 …… **S6**

8- Figure S4 …… **S6**

9- Figure S5 …… **S7**

10- Figure S6 …… **S7**

11- Figure S7 …… **S8**

12- Figure S8 …… **S8**

13- Characterization of products …… **S9**

14- NMR copies …… **S12**

15- IR copies …… **S28**

1- Materials and characterization methods

Reagents

Reagents (≥ 97% purity) and solvents (≥ 99% purity) used in this work were all purchased from commercial suppliers (Merck, TCI, Apollo Scientific, Fluorochem, Scharlab) and used as received unless otherwise indicated. The acceptor 9,10 diphenylanthracene (**DPA**) was commercially available from Sigma Aldrich, 99% purity. **BOPHY-1** was synthesized following a previous procedure.[1] Sensitizer **silica@BOPHY-2** as well as several diphenyl derivatives were synthesized as described (*vide infra*).

Characterization

Determination of purity and structure confirmation of the literature known products was performed by ^{1}H, ^{13}C, ^{19}F and ^{11}B NMR and low-resolution mass spectrometry (LRMS)–LRMS measurements were replaced by high-resolution mass spectrometry (HRMS) in case of unknown products. NMR spectral data were collected on a Bruker Advance 400 (400 MHz for ^{1}H; 101 MHz for ^{13}C; 376 MHz for ^{19}F; 128 MHz for ^{11}B; 162 MHz for ^{31}P) spectrometer at 20 °C. Chemical shifts are reported in δ/ppm, coupling constants J are given in Hertz. Solvent residual peaks were used as internal standard for all NMR measurements. The quantification of ^{1}H cores was obtained from integrations of appropriate resonance signals. Abbreviations used in NMR spectra: s = singlet, d = doublet, t = triplet, q = quartet, m = multiplet, bs = broad singlet, dd = doublet of doublet, ddd = doublet of doublet of doublet. HRMS was carried out was performed in the mass facility of Instituto de Tecnología Química (ITQ). LRMS was carried out on an HP 6890 Series GC System with Agilent 5973 Network Mass Selective Detector and H_2 as carrier gas. Abbreviations used in MS spectra: M—molar mass of target compound, EI—electron impact ionization, ESI—electrospray ionization.

Ultraviolet–visible spectra (UV–Vis) of the samples were obtained by a CARY 60 spectrophotometer. The samples were placed into quartz cells of 1 cm path length. Compound concentrations were fixed as indicated. The UV-Vis in solid phase was obtained by diffuse reflectance in order to check whether the absorption band was comparable with that in solution.

2- Synthetic methods

General procedure for biphenyls synthesis: In a 50 mL bottom flask equipped with a magnetic stir bar and a reflux condenser, 1 mmol of the corresponding iodobenzene, 3 mmol of the corresponding styrene, 20 mol% Pd-Cs(3)X and 30 mL mesitylene (1 M) were added. The reaction was then heated at 175°C at reflux in open air. Aliquots of the supernatant (50 µL) were periodically withdrawn after stopping the stirring for a few seconds, and diluted in toluene solution with the internal standard, to be analyzed by GC. After 2 h, the reaction mixture was cooled, 50 mL of water was added and the mixture was extracted with 50 mL of ethyl acetate (three times), washed with brine and dried over magnesium sulfate. It was then purified by flash chromatography in a mixture of hexane: ethyl acetate (4:1) as eluent. Once separated it was concentrated under vacuum obtaining the corresponding pure biphenyl for the following reaction.

Synthesis of silica@BOPHY-2 sensitizer: In a round bottom flask equipped with a magnetic bar, add 0.17 mmol of **BOPHY-1**, 0.17 mmol of styrene (1 eq), $Pd(OAc)_2$ (1.7-10-4 mmol, 0.1% mol), TPABr (0.0255 mmol, 15% mol), K_2CO_3(0.255 mmol, 1.5 eq) and 1.7 mL of DMF (0. 1 M), stirred for 2 hours at 130°C, once all the styrene is consumed, allowed to cool to room temperature, 5 mL of water is added and extracted with ethyl acetate (3 x 5 mL), the organic phase is washed twice with water (5 mL) and finally washed with brine, this organic phase is dried with $MgSO_4$, filtered and concentrated under vacuum (84% yield **BOPHY-2**). The crude of the reaction is used for the next step, where AIBN ($1.7 \cdot 10^{-3}$ mmol, 1%mol), 200 mg of 3-Mercaptopropyl-functionalized silica gel 200-400 mesh (1.2 mmol/g loading) and 1.7 mL of toluene (0.1M) are added, put at 50°C for 16 h. Once the time has passed, the solid of the reaction is put in an envelope of filter paper and by means of a Soxhlet system it is left 6 hours making cycles with toluene as solvent to drag everything that is without anchoring to the solid. The solid is left to dry at 100 ºC for 16 hours and is

[1] Tamgho, I.-S.; Hasheminasab, A.; Engle, J. T.; Nemykin, V. N.; Ziegler, C. J. A new highly fluorescent and symmetric pyrrole–BF2 chromophore: BOPHY. *J. Am. Chem. Soc.* **2014**, *136*, 5623−5626.

measured by elemental analysis to know the percentage of organic matter anchored to the solid. The weight percentage (wt%) of **BOPHY-2** anchored to silica was obtained through an elemental analysis study of the sample silica@**BOPHY-2**, where 0.52% of Nitrogen was observed in the sample. As 4 Nitrogen atoms are in each molecule of **BOPHY-2**, we calculated the amount of **BOPHY-2** anchored to functionalized silica, obtaining a value of 5.3 wt%.

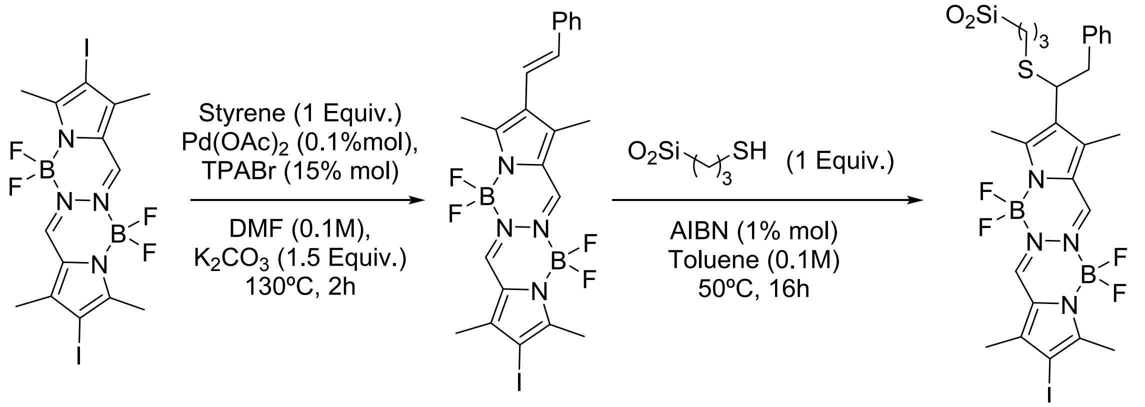

BOPHY-1 **BOPHY-2** **silica@BOPHY-2**

General procedure A for the coupling reactions (batch): In a 4mL quartz cuvette with a magnetic stirrer, 1 eq of 4-bromobenzaldehyde, **BOPHY-2** (170 µg, 0.3 µmol, 10^{-4} M, 0.01 eq), **DPA** (1 mg, 3 µmol, 10^{-3} M, 0.1 eq) and the corresponding equivalents of trapping agent were dissolved in 3 mL total volume of solvent mixture composed by ACN/DMA (4:1, v:v), then 1 eq of dodecanenitrile as internal standard was added and the cuvette was sealed under Ar atmosphere, partially submerged in a cold water bath and irradiated by an external diode laser pointer (λ_{exc} = 445 nm ± 10) through one face of the cuvette . The photoreaction was followed by TLC on commercial SiO_2-coated aluminium plates (DC60 F254, Merck). Visualization was done by UV-light 254 nm. After 4h, the crude of the reaction was extracted with ethyl acetate, washed with brine and evaporated under vacuum. Finally, the product was purified by high performance liquid chromatography (HPLC) using ACN/H_2O 90/10 as eluent. Yield products were estimated as: [conversion × selectivity]/mass balance. Also, isolated yield product was obtained.

The same conditions were used in homogeneous continuous flow conditions, extrapolating all the amounts to a total volume of 50 mL.

General procedure B for the coupling reactions (continuous flow): In a 100mL flask with a magnetic stirrer inside, and which is connected to a flow system and to a two-cap vial with the **silica@BOPHY-2**, 1 eq of aryl halide, **DPA** (5%) and the corresponding equivalents of trapping agent were placed in a solvent mixture composed by ACN/DMA (4:1, v:v), then the flask was sealed under Ar atmosphere, and the two-cap vial was irradiated by an external diode laser pointer (λ_{exc} = 445 nm ± 10) pointing to the hybrid material. The photoreaction was followed by TLC on commercial SiO_2-coated aluminium plates (DC60 F254, Merck). Visualization was done by UV-light 254 nm. After 22h, the crude of the reaction was extracted with ethyl acetate, washed with brine and evaporated under vacuum. Finally, the product was purified by silica gel flash chromatography using Hexane/Ethyl acetate 100:1 as eluent. Yield products were obtained as isolated yields.

3- Irradiation light source

The coupling reactions were carried out using a blue diode laser pointer with a real power of 2000 mW (λ_{exc} = 445 ± 10 nm, beam diameter of 5 mm x 3 mm, intensity of 1.64 mJ/cm^2) has been purchased from ®TorLaser.

4- Transient absorption spectroscopy (TAS)

The LP980–KS Laser Flash Photolysis Spectrometer (from Edinburgh Instruments) is a combined system for the measurement of laser induced transient absorption, emission kinetics and spectra, with the ability to

automatically convert and fully analyze the kinetic and spectral information. The probe pulse is longer than the recorded time window of a measurement, and a monochromator (TMS302-A, grating 150 lines mm−1) disperses the probe light after it passed the sample. The probe light can be then passed on to a PMT detector (spectral S5 range 200–870 nm) to obtain the temporal resolved picture. All components are controlled by the software L900 provided by Edinburgh.

For our delayed emission measurements, the probe shutter is closed so that no light from the Xe lamp is exciting the sample, and the laser is only used as a light source. To photolyze our samples, a 485 nm monowavelength was employed, ensuring that only the BOPHY chromophore absorbs the excited photons. The data have been acquired as an average of several shots to improve the signal-to-noise ratio.

5- Table S1

Table S1. Optimization of reaction between 4-bromobenzaldehyde and 1,1-diphenylethylene. Isolated yields by flash column chromatography.

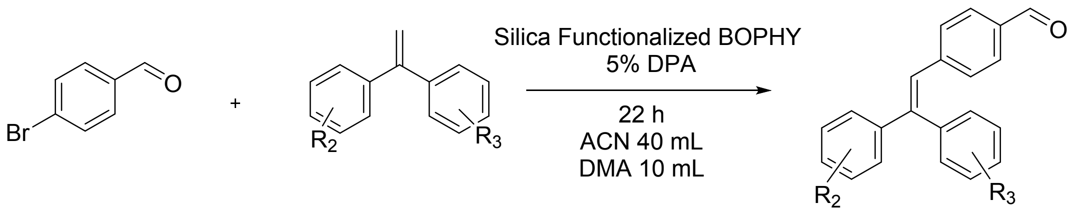

Entry	Substrate (eq)	Time (h)	Trapping (eq)	DPA (% mol)	Selectivity (%)	Yield (%)
1	500 mg	6h	2 eq	30	100	35.1
2	500 mg	18h	2 eq	30	100	44.6
3	*250 mg*	*18h*	*5 eq*	*5*	*100*	*48.9/37.7/28.7*[a]
**4**	**100 mg**	**22h**	**10 eq**	**5**	**100**	**59**
5	100 mg	22h	10 eq	0	0	0[b]
6	100 mg	22h	10 eq	5	0	0[c]
7	100 mg	22h	10 eq	5	100	13.2[d]

a) Photocatalyst reused for the experiments.
Control experiments:
b) Without **DPA**
c) Undoped silica without **BOPHY-2**
d) Undoped silica, **BOPHY-2** in solution

6- Continuous-flow set-up

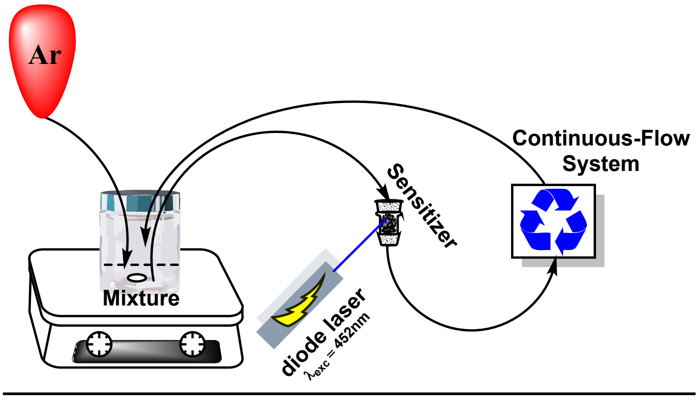

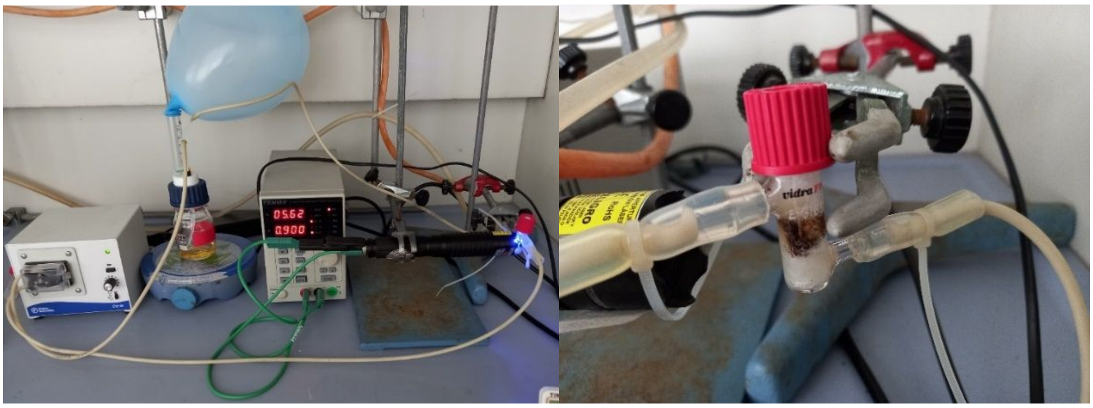

Figure S1. Photograph of the set-up for the photocatalytic coupling reaction by TTA technology in continuous-flow conditions.

To construct the pellets of the **silica@BOPHY-2** hybrid material, we first form a big pellet by putting a part of this material in a die, where a pressure of 10 tons is exerted for 5 minutes. Then, this material is poured into a mortar that it is broken little by little introducing it into a sieve whose size was between 0.2 and 0.6 µm, this operation is repeated several times until obtaining an amount that allows us to load the reactor, thus obtaining our pellets of 0.2-0.6 µm size.

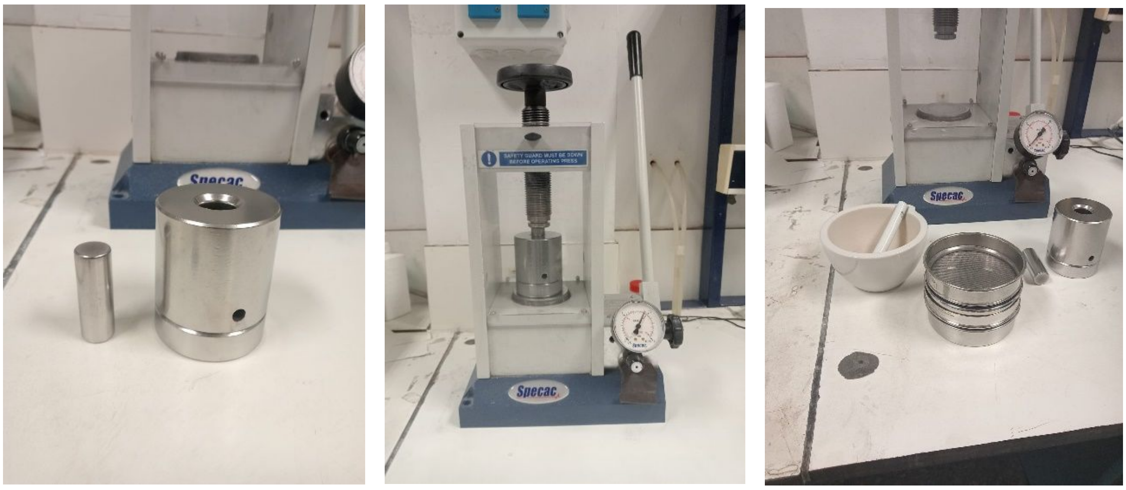

1. Die   2. Die in press   3. Morter and sieve

Figure S2. Photograph of the procedure for fabricating the corresponding pellets.

7- **Figure S3**

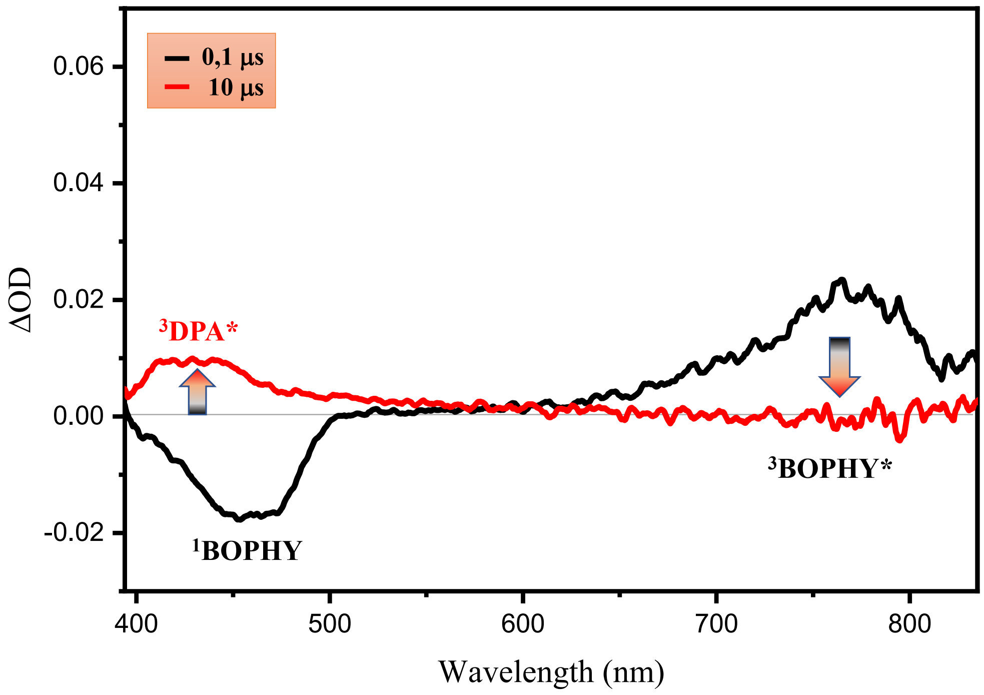

Figure S3. Transient absorption spectra of **BOPHY-2** (0.01 mM) and **DPA** (0.1 mM) in N_2 ACN/DMA (4/1 v/v) solution (λ_{exc} = 450 nm)

8- **Figure S4**

To prepare the corresponding dispersed solution, 20 mg of the material (silica@BOPHY-2) was added to 3 mL of ACN/DMA (4/1) and sonicated for 30 minutes. Then, an aliquot of 1 mL was taken, and the total volume of 3 mL was completed by 2 mL of ACN/DMA (4/1). Then, 1 mg of DPA was added to the 3 mL dispersed solution The whole mixture was degassed by N_2 for 10 minutes.

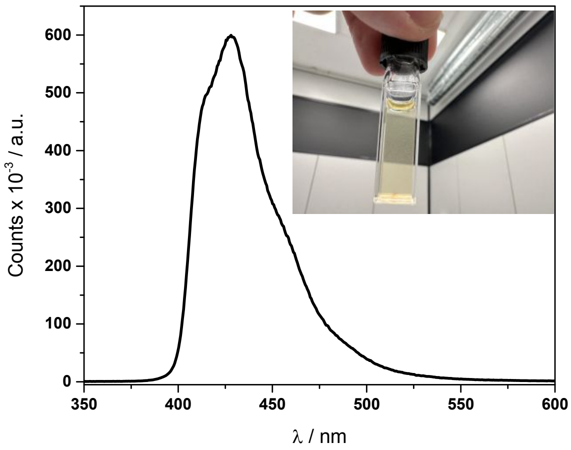

Figure S4. Emission spectrum of silica@BOPHY-2 and DPA (1 mg) in 3 mL of an anaerobic ACN/DMA dispersion recorded 2 µs after the laser pulse (λ_{exc} = 450 nm).

9- Figure S5

To directly observe the radical cation of **DPA** which is generated by an electron transfer with an appropriate electron acceptor, laser flash photolysis (LFP) study has been carried out. After irradiation (λexc = 355 nm) to a DPA solution in the presence of 2-acetyl-5-chlorothiophene, typical transient absorption of the DPA radical cation was observed based on literature data. [2]

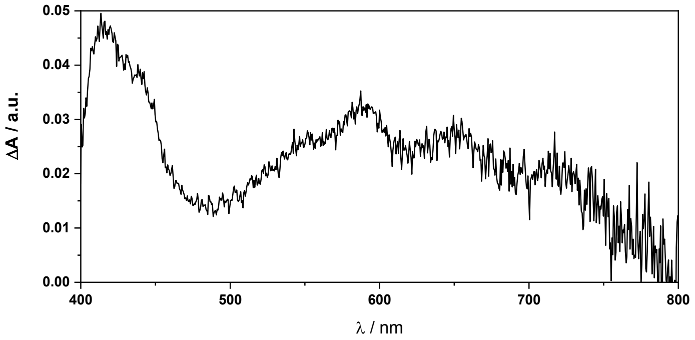

Figure S5. Laser flash photolysis (λ_{exc} = 355 nm) spectrum of **DPA** (0.1 mM) in the presence of 2-acetyl-5-chlorothiophene (1 mM) in degassed ACN/DMA (4/1 v/v) solution recorded 100 ns after the pulse laser.

10- Figure S6

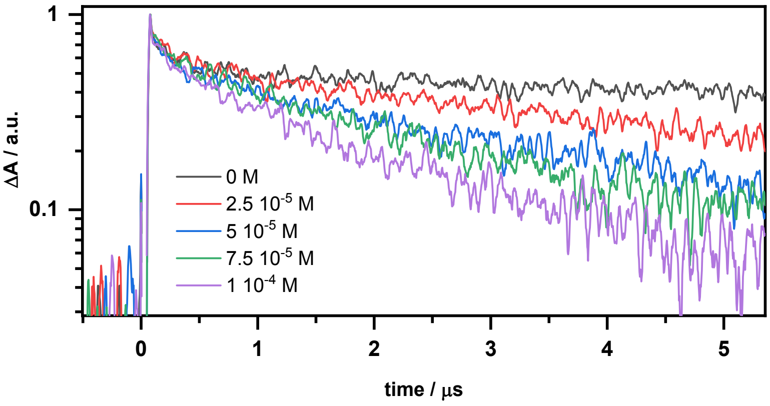

Figure S6. Decays monitored at 700 nm after 450 nm laser excitation of **BOPHY-2** (0.01 mM) in anaerobic ACN/DMA solution in the presence of increasing amounts of **DPA**.

[2] M. S. Workentin, et. al. *J. Am. Chem. Soc.* **1994**, *116*, 8279.

11- Figure S7

We have performed absorptivity measurements. The extinction coefficient of BOPHY-2 has been found to be 7189 $M^{-1}cm^{-1}$ (figure S7A). In this context, comparison of the UV-Vis spectra (figure S7B) of silica-BOPHY-2 in dispersed phase, silica@BOPHY-2 in solid phase and BOPHY-2 in solution have been recorded and the absorbances at excitation wavelength are quite similar. For the dispersed solution, 3,27 mg of silica@BOPHY-2 were placed in a 1 cm pathlength cuvette, adding 3 mL of a mixture of ACN/DMA (4/1, v/v). The absorbance after subtracting the silica baseline (dispersed solution alone) (Figure S7B, grey line) was 0.64681 at 450 nm. Since the BOPHY-2 was found to be 5.3 wt%, the molar extinction coefficient was estimated as 6325 $M^{-1}cm^{-1}$. Therefore, we believe that studies in batch or flow conditions could be comparable.

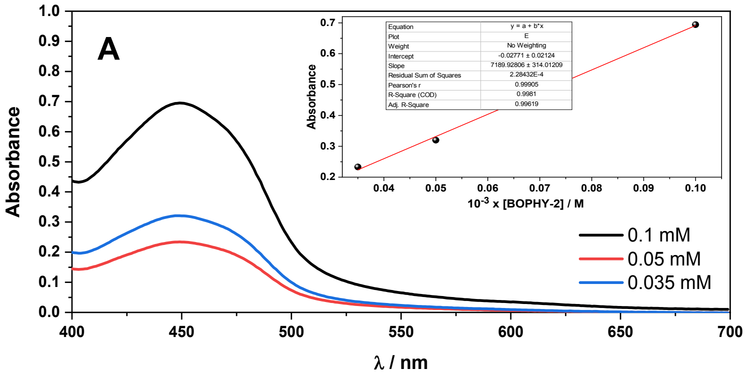

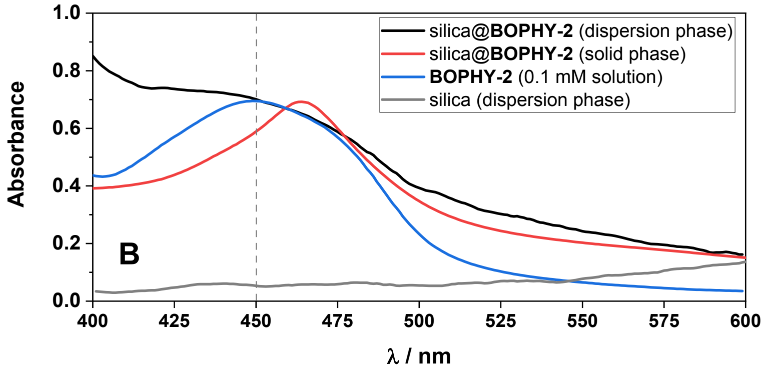

12- Figure S8

The retention times (RT) of both the internal standard and DPA are 9.6 min and 22.3 min, respectively. Considering the peak areas, DPA is not consumed at all.

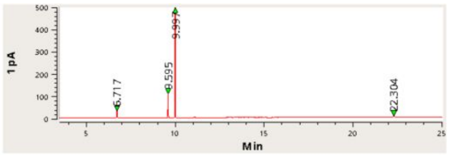


```
[FID1A] Area Percent Report

Sample Name        :JCS152-Olan    Sample Type   :sample
Injection Source   :turret         Location      :1
Amount             :1              Volume        :as method
```

PEAK#	RT[min]	Width[min]	Area	Height	Area%	Name
1	2.069	0.041	199310	81335.4	96	
2	2.917	0.038	6721.67	2406.4	3	
3	6.717	0.029	56.8991	31.046	0	
4	9.595	0.027	182.158	108.529	0	
5	9.997	0.030	1001.9	532.651	0	
6	22.304	0.064	26.5445	6.28257	0	
Sum:			207298.812183	84420.316868	100	

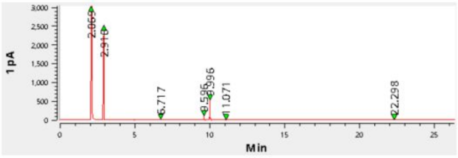


```
[FID1A] Area Percent Report

Sample Name        :JCS152-O2de           Sample Type   :sample
Injection Source   :turret                Location      :2
Volume             :as method ng/uL
```

PEAK#	Type	RT[min]	Width[min]	Area	Height	Area%	Name
1	BB	2.069	0.040	194208	80069.3	96	
2	BB	2.916	0.049	6935.33	2523.01	3	
3	BB	6.717	0.030	36.8273	19.9356	0	
4	BB	9.596	0.026	189.709	114.095	0	
5	BB	9.996	0.030	997.422	530.015	0	
6	BB	11.071	0.029	13.1056	6.92506	0	
7	BB	22.298	0.065	28.8895	6.75119	0	
Sum:				202409.290602	83270.043099	100	

Figure S7. GC spectra of irradiation of 2-acetyl-5-chlorothiophene and 1,1-diphenylethylene following the general procedure B. *Left*: before irradiation; *Right*: after irradiation.

13- Characterization of products

BOPHY-2 ((*E*)-(2-bophyvinyl)benzene)

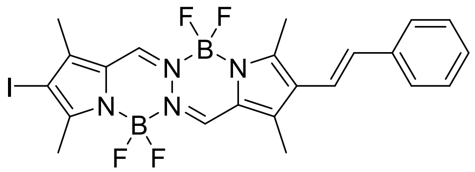

^{1}H NMR (300 MHz, $CDCl_3$) δ 7.95 (s, 2H), 7.28 – 7.21 (m, 5H), 7.04 (d, J = 14.0 Hz, 1H), 6.70 (d, J = 14.0 Hz, 1H), 2.89 (s, 6H), 2.81 (s, 6H).
^{13}C NMR (75 MHz, $CDCl_3$) δ 161.56 (s), 136.16 (s), 134.90 (s), 127.95 (s), 127.77 (s), 127.25 (s), 127.20 (s), 125.08 (s), 105.48 (s), 59.38 (s), 35.48 (s), 30.43 (s), 28.68 (s), 13.17 (s).
^{19}F NMR (282 MHz, $CDCl_3$) δ -141.78 – -142.77 (m).
IR (cm^{-1}): 3316.9 (m), 2918.7 (m), 2364.3 (m), 1718.3 (i), 1662.4 (i), 1579.4 (vi).

SiO_2@BOPHY-2 ((1-bophy-1-SiO_2ethyl)benzene)

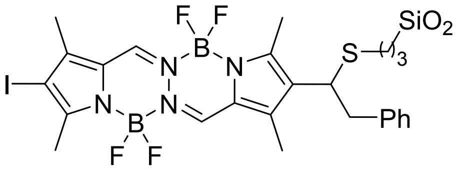

IR (cm^{-1}): 2359.5 (s), 1978.6 (s), 1617.0 (s), 1044.3 (vi).
^{13}C NMR (101 MHz) δ 53.63, 41.37, 22.34, 11.65.
^{11}B NMR (128 MHz) δ -2.35.
UV-Vis: Absorption band (460 nm).
EA: N (%) 0.494, C (%) 7.798, H (%) 1.479, S (%) 3.463.

1-Methyl-3-(1-phenylvinyl)benzene

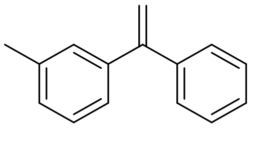

The compound was prepared according to the general procedure for biphenyls synthesis using 3 mmol of iodobenzene and 9 mmol of the 3-methylstyrene as starting materials, obtaining 315 mg of 1-methyl-3-(1-phenylvinyl)benzene (54% isolated yield).

^{1}H NMR (300 MHz, $CDCl_3$) δ 7.52 – 7.38 (m, 5H), 7.39 – 7.19 (m, 4H), 5.55 (s, 2H), 2.45 (s, 3H).
^{13}C NMR (75 MHz, $CDCl_3$) δ 150.28, 141.71, 141.59, 137.79, 129.04, 128.56, 128.36, 128.22, 128.15, 127.74, 125.55, 114.21, 21.51.
GC-MS: Calculated: 194.11; Experimental: 194.1.

2-(1-Phenylvinyl)aniline

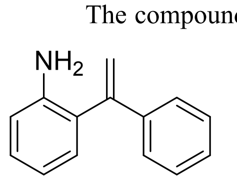

The compound was prepared according to the general procedure for biphenyls synthesis using 3 mmol of iodobenzene and 9 mmol of the *o*-vinilaniline as a starting materials, obtaining 234 mg of 2-(1-phenylvinyl)aniline (40 % isolated yield).
^{1}H NMR (401 MHz, $CDCl_3$) δ 7.43 – 7.36 (m, 2H), 7.37 – 7.28 (m, 3H), 7.23 – 7.07 (m, 2H), 6.82 (td, *J* = 7.4, 1.1 Hz, 1H), 6.73 (dd, *J* = 8.0, 1.2 Hz, 1H), 5.82 (d, *J* = 1.5 Hz, 1H), 5.38 (d, *J* = 1.4 Hz, 1H), 3.62 (s, 2H).
^{13}C NMR (101 MHz, $CDCl_3$) δ 147.16, 143.77, 139.70, 130.88, 128.82, 128.62, 128.14, 127.49, 126.69, 118.52, 116.22, 115.75.
GC-MS: Calculated: 195.10; Experimental: 195.0.

1,2-Dimethyl-4-(1-phenylvinyl)benzene

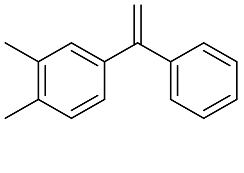

The compound was prepared according to the general procedure for biphenyls synthesis using 3 mmol of iodobenzene and 9 mmol of the 1,2-dimethylstyrene as a starting materials, obtaining 363 mg of 1,2-dimethyl-4-(1-phenylvinyl)benzene (58 % isolated yield).

^{1}H NMR (401 MHz, $CDCl_3$) δ 7.42 – 7.30 (m, 5H), 7.18 – 7.06 (m, 3H), 5.45 (d, *J* = 1.4 Hz, 1H), 5.42 (d, *J* = 1.4 Hz, 1H), 2.31 (s, 3H), 2.28 (s, 3H).

^{13}C NMR (101 MHz, $CDCl_3$) δ 150.07, 141.82, 139.15, 136.32, 136.24, 129.48, 129.48, 128.34, 128.14, 127.63, 125.80, 113.58, 19.83, 19.54.

GC-MS: Calculated: 194.11; Experimental: 194.1.

4-(2,2-Diphenylvinyl)benzaldehyde

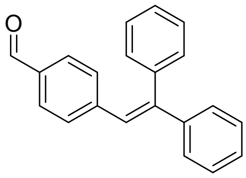

The compound was prepared according to the general procedure B using 4-bromobenzaldehyde (100 mg, 0.5 mmol, 1.0 eq), 1,1-diphenylethylene (0.92 mL, 5 mmol, 10.0 eq), **DPA** (8.25 mg, 25 µmol, 0.05 eq) and **silica-BOPHY-2** (64 mg, 0.012 eq of sensitizer). The desired product was obtained with a 59% of isolated yield (83.2 mg).

^{1}H NMR (400 MHz, CD_3CN) δ 9.88 (s, 1H), 7.65 (d, J = 8.1 Hz, 2H), 7.43 – 7.32 (m, 8H), 7.23– 7.16 (m, 4H), 7.13 (s, 1H) ppm.

^{13}C NMR (101 MHz, CD_3CN) δ 192.5, 146.2, 144.3, 143.3, 140.5, 135.3, 130.6, 130.6, 129.7, 129.5, 129.0, 128.8, 128.1, 127.5 ppm.

Spectral data were consistent with literature (see for instance: *Tetrahedron*, **2011,** 67, 250).

1-(5-(2,2-Diphenylvinyl)thiophen-2-yl)ethanone

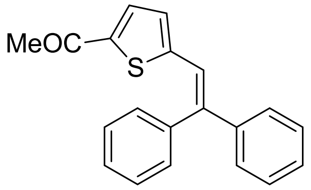

The compound was prepared according to the general procedure B using 5-chloro-2-acetylthiophene (100 mg, 0.5 mmol, 1.0 eq), 1,1-Diphenylethylene (0.92 mL, 5 mmol, 10.0 equiv.), **DPA** (8.25 mg, 25 µmol, 0.05 eq) and **silica-BOPHY-2** (64 mg, 0.012 eq). The desired product was obtained with a 43% (42.9%) of isolated yield (65.3 mg)

^{1}H NMR (400 MHz, CD_3CN) δ 7.58 – 7.51 (m, 4H), 7.41 (s, 1H), 7.39 – 7.30 (m, 5H), 7.26 (dd, J = 6.7, 2.9 Hz, 2H), 7.08 (d, J = 4.0 Hz, 1H), 2.39 (s, 4H).

^{13}C NMR (101 MHz, CD_3CN) δ 190.6, 133.5, 131.5, 130.7, 129.6, 129.5, 129.1, 127.8, 121.5, 26.7.

GC-MS (EI): m/z (relative intensity): 304 (100) [M+•], 289 (50), 228 (40), 202 (13), 152 (10), 43 (16).

Spectral data were consistent with literature data (see for instance: *Nat Catal,* **2021**, 4, 293)

(*Z/E*)-1-(5-(2-Phenyl-2-(m-tolyl)vinyl)thiophen-2-yl)ethenone.

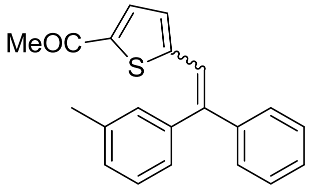

The compound was prepared according to the general procedure B using 5-chloro-2-acetylthiophene (100 mg, 0.6 mmol, 1.0 eq), 1-methyl-3-(1-phenylvinyl)benzene (600 mg, 300 mmol, 5.0 eq), **DPA** (8.25 mg, 25 µmol, 0.05 eq) and **silica-BOPHY-2** (64 mg, 0.012 eq). The desired product was obtained in a 1:1 isomeric mixture with a 39% yield (74.9 mg).

^{1}H NMR (400 MHz, CD_3CN) δ 7.56 – 7.54 (m, 4H), 7.39-7.31 (m, 7H), 7.29 (s, 1H), 7.28 (s, 1H), 7.24-7.20 (m, 4H), 7.16-7.10 (m, 5H), 2.39 (s, 3H), 2.38 (s, 3H)

^{13}C NMR (101 MHz, CD_3CN) δ 191.8, 191.7, 149.8, 144.8, 144.4, 142.1, 140.5, 139.8, 139.7, 139.1, 138.1, 133.5, 131.5, 131.4, 131.0, 130.6, 130.6, 130.5, 130.2, 129.9, 129.5, 129.4, 129.4, 129.3, 129.1, 128.7, 128.4, 127.8, 127.8, 127.6, 127.1, 125.6, 125.1, 121.3, 118.3, 116.6, 26.7, 26.7, 21.4, 21.4.

GC-MS (EI): m/z (relative intensity): 318 [M+•], 303 (25), 209 (56), 192 (47), 179 (20), 147 (54), 119 (35)

HRMS (EI): m/z $(M+H)^+$ = calcd. for $C_{21}H_{18}OS$: 319.1151, found: 319.1163.

(E)-1-(5-(2-(2-aminophenyl)-2-phenylvinyl)thiophen-2-yl)ethan-1-one

1-(5-(2-Amino-3-(1-phenylvinyl)phenyl)thiophen-2-yl)ethan-1-one

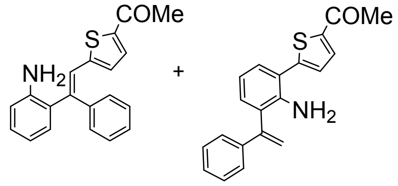

The compounds were prepared according to the general procedure B using 5-chloro-2-acetylthiophene (100 mg, 0.6 mmol, 1.0 equiv.), 2 -(1-phenylvinyl)aniline (614 mg, 300 mmol, 5.0 equiv.), **DPA** (8.25 mg, 25 μmol, 0.05 eq) and **silica-BOPHY-2** (64 mg, 0.012 eq). The desired product was obtained in a 1:1 isomeric mixture with a 38% yield (72.6 mg).

^{1}H NMR (400 MHz, CD_3CN) δ 7.60-7.59 (d, J = 4Hz, 1H), 7.53 (m, 1H), 7.45-7.43 (m, 2H), 7.36-7.29 (m, 5H), 7.17-7.16 (d, J = 4Hz, 2H), 6.94-6.92 (dd, J = 6.7, 1.2Hz, 1H), 6.84 (s, 1H), 4.08-3.96 (m, 2H), 2.40 (s, 3H)

^{1}H NMR (400 MHz, CD_3CN) δ 7.77-7.76 (d, J = 4Hz, 1H), 7.38-7.28 (m, 6H), 7.27-7.26 (d, J = 3.9 Hz, 2H), 7.11-7.09 (dd, J = 7.4, 1.6 Hz, 1H), 6.81-6.78 (t, J = 7.6 Hz, 1H), 5.89-5.88 (d, J = 1.3 Hz), 5.33-5.33 (d, J = 1.3 Hz, 1H), 4.17 (m, 2H), 2.51 (s, 3H)

^{13}C NMR (101 MHz, CD_3CN) δ 191.6, 149.3, 145.9, 144.6, 140.8, 140.4, 135.1, 133.1, 131.1, 130.9, 130.6, 129.2, 128.9, 127.1, 127.1, 122.7, 119.1, 23.0.

^{13}C NMR (101 MHz, CD_3CN) δ 191.7, 150.7, 147.9, 144.4, 143.2, 140.3, 134.7, 132.4, 131.1, 129.6, 129.2, 128.3, 127.4, 119.7, 118.5, 117.9, 117.4, 26.8.

GC-MS (EI): m/z (relative intensity): 319 (92) [M+•], 318 (100), 304, (42), 276 (29), 262 (10), 103 (11).

GC-MS (EI): m/z (relative intensity): 319 (100) [M+•], 318 (15), 276 (29), 243 (45).

HRMS (EI): m/z $(M+H)^+$ = calcd. for $C_{20}H_{17}NOS$: 320.1104, found: 320.1102.

(*Z/E*)-1-(5-(2-(3,4-Dimethylphenyl)-2-phenylvinyl)thiophen-2-yl)ethan-1-one

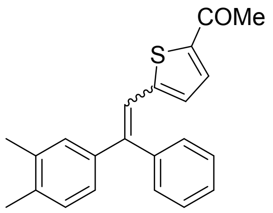

The compound was prepared according to the general procedure B using 5-chloro-2-acetylthiophene (100 mg, 0.6 mmol, 1.0 equiv.), 1,2-dimethyl-4-(1-phenylvinyl)benzene-methane (520 mg, 300 mmol, 5.0 equiv.), **DPA** (8.25 mg, 25 μmol, 0.05 eq) and **silica-BOPHY-2** (64 mg, 0.012 eq). The desired product was obtained in a 1:1 isomeric mixture with an 9% of isolated yield (18.9 mg).

^{1}H NMR (400 MHz, CD_3CN) δ 7.57-7.56 (d, J = 4Hz, 2H), 7.54-7.52 (m, 1H), 7.36-7.33 (m, 6H), 7.08-7.07 (d, J = 4Hz), 7- 6.97 (m, 3H), 2.39 (s, 3H), 2.37 (s, 3H), 2.29 (s, 3H).

^{13}C NMR (101 MHz, CD_3CN) δ 191.4, 149.6, 144.6, 144.0, 142.1, 138.6, 137.7, 133.1, 131.3, 131.1, 131.0, 130.2, 129.0, 128.7, 127.6, 127.5, 120.9, 26.3, 19.41, 19.4.

HRMS (EI): m/z $(M+H)^+$ = calcd. for $C_{22}H_{20}OS$: 333.1308, found: 333.1304.

5-(2,2-Diphenylvinyl)thiophene-2-carbonitrile

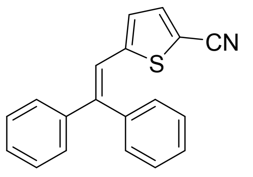

The compound was prepared according to the general procedure B using 5-Bromo-2-carbonitrile (112 mg, 0.6 mmol, 1.0 equiv.), 1,1-diphenylethylene (0.92 mL, 5 mmol, 10.0 equiv.), **DPA** (8.25 mg, 25 μmol, 0.05 eq) and **silica-BOPHY-2** (64 mg, 0.012 eq). The desired product was obtained with a 49% yield (85.2 mg).

^{1}H NMR (400 MHz, Acetone) δ 7.63-7.62 (d, J= 4 Hz, 1H), 7.60-7.58 (m, 4H), 7.42-7.32 (m, 5H), 7.27-7.25 (m, 2H), 7.24 (dd, 4, 0.6 Hz, 1H)

^{13}C NMR (101 MHz, Acetone) δ 149.06, 144.97, 141.28, 139.13, 137.65, 130.82, 130.59, 130.52, 129.83, 129.30, 129.15, 127.69, 120.43, 114.86, 109.26, 29.76.

14- NMR Copies

^{1}H (300 MHz, $CDCl_3$)

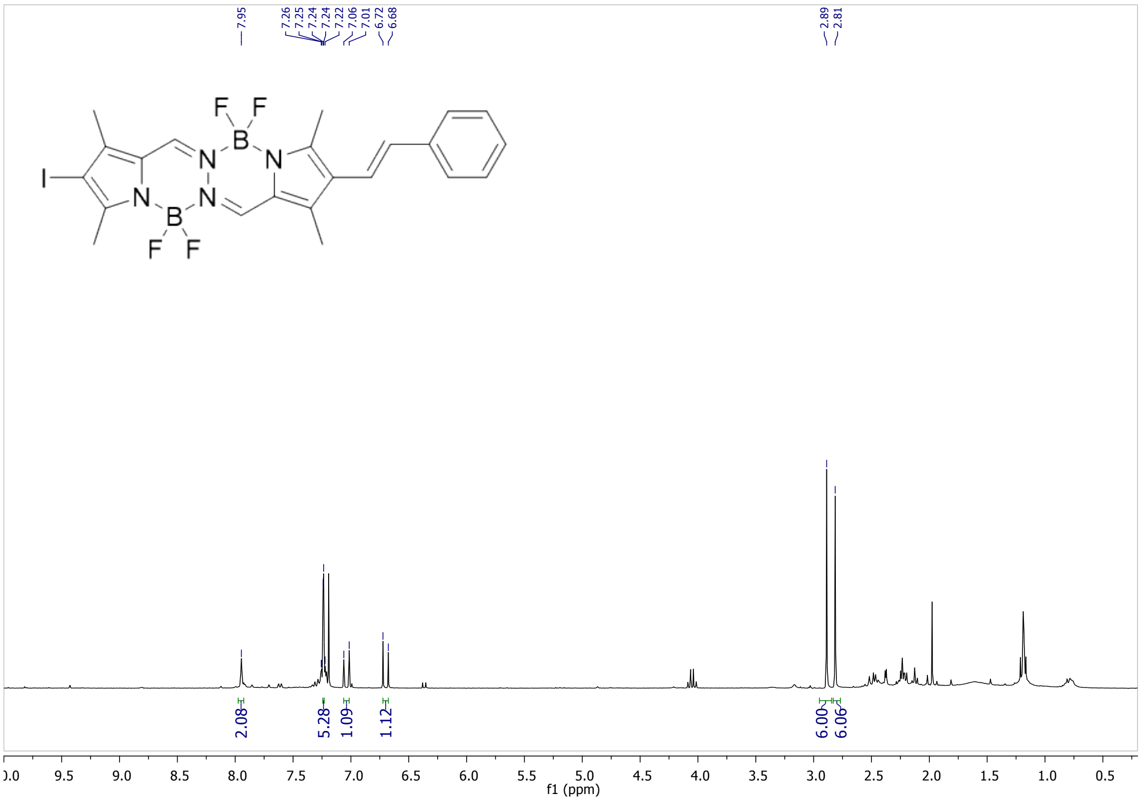

^{13}C NMR (75 MHz, $CDCl_3$)

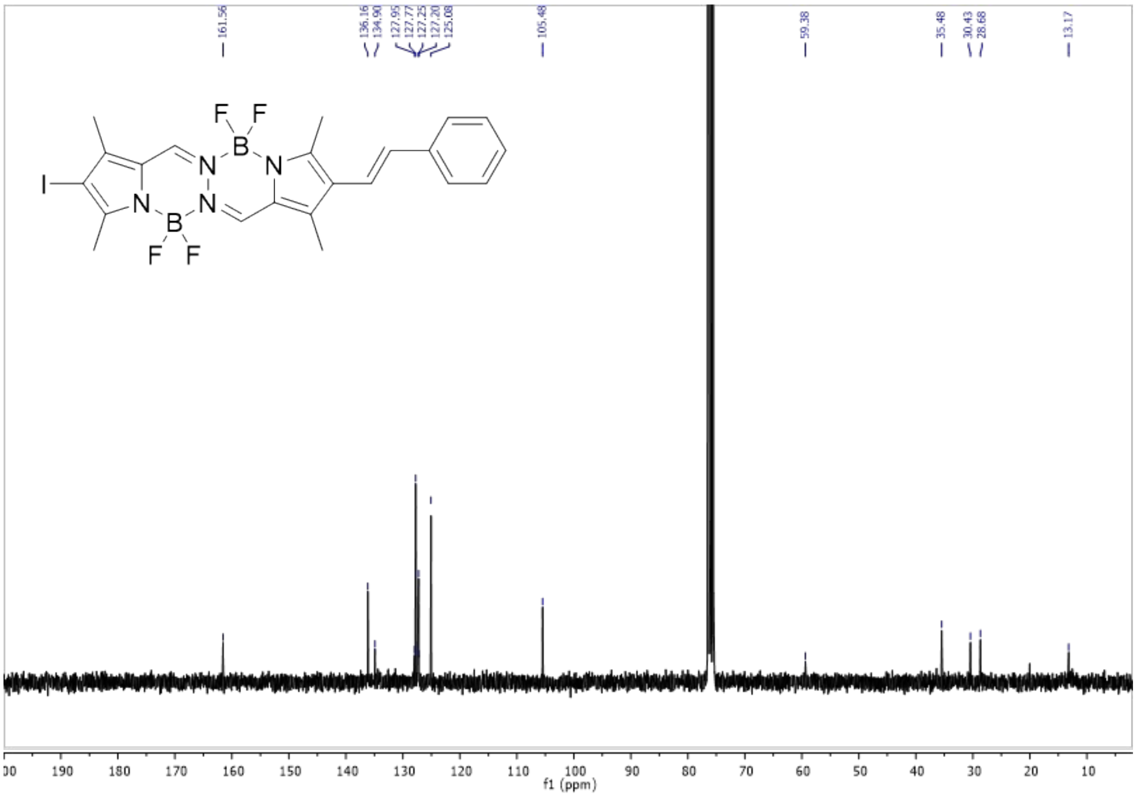

DEPT (75 MHz, $CDCl_3$)

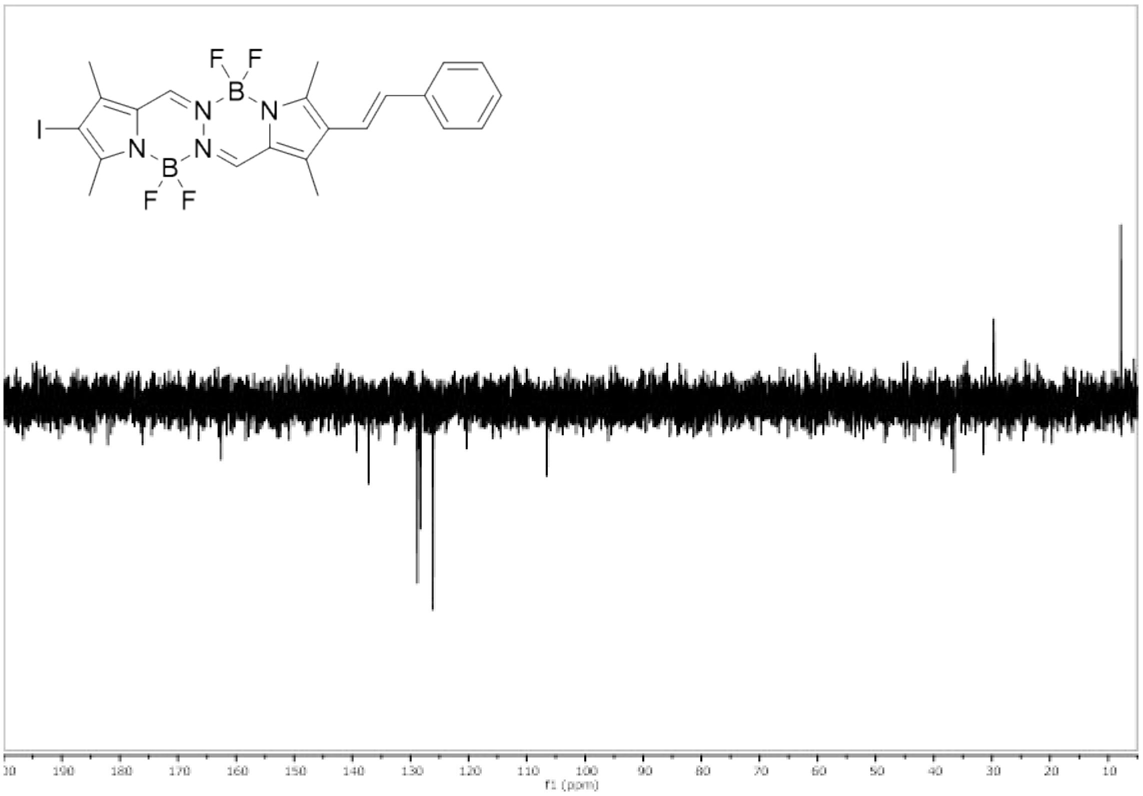

^{19}F NMR (282 MHz, $CDCl_3$)

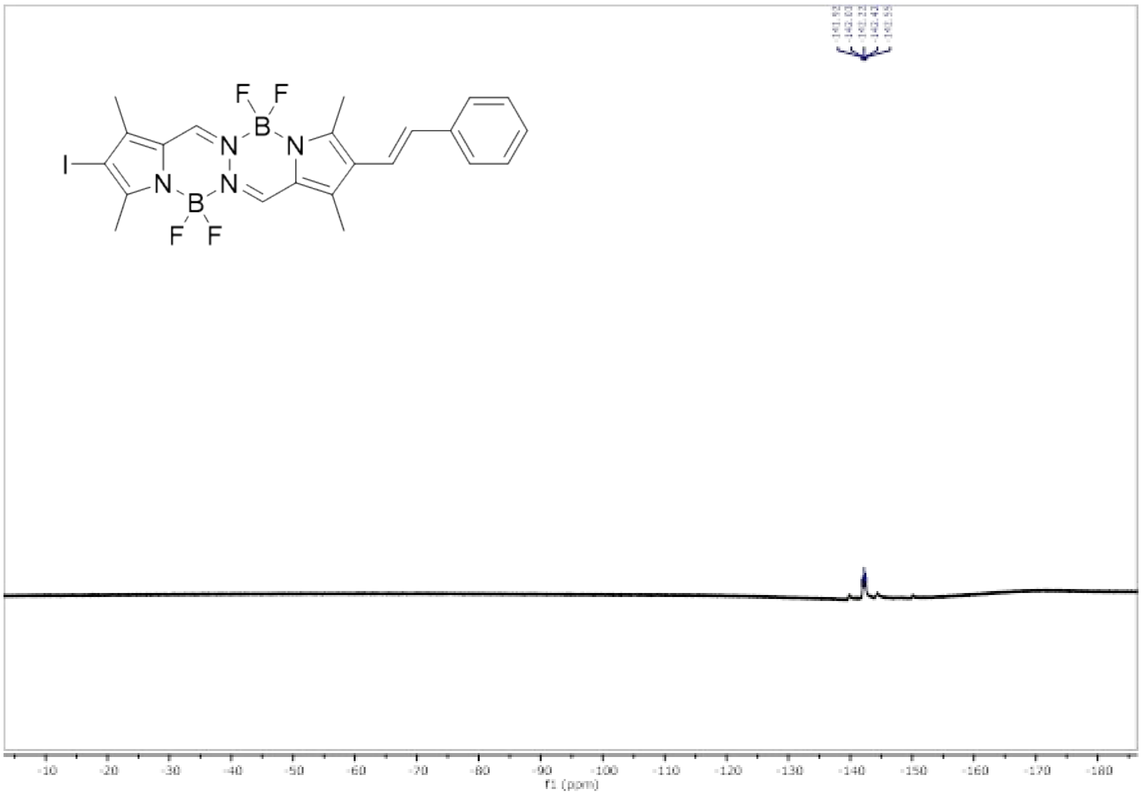

^{13}C NMR (101 MHz)

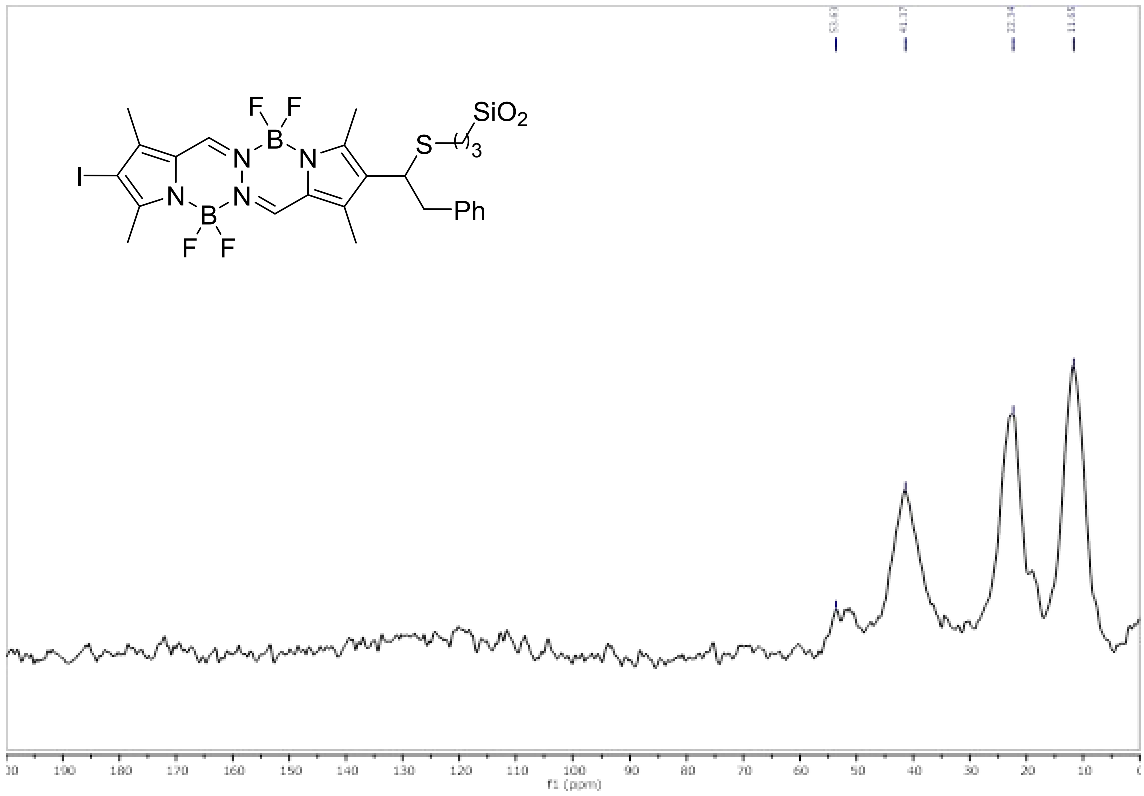

^{11}B NMR (128 MHz)

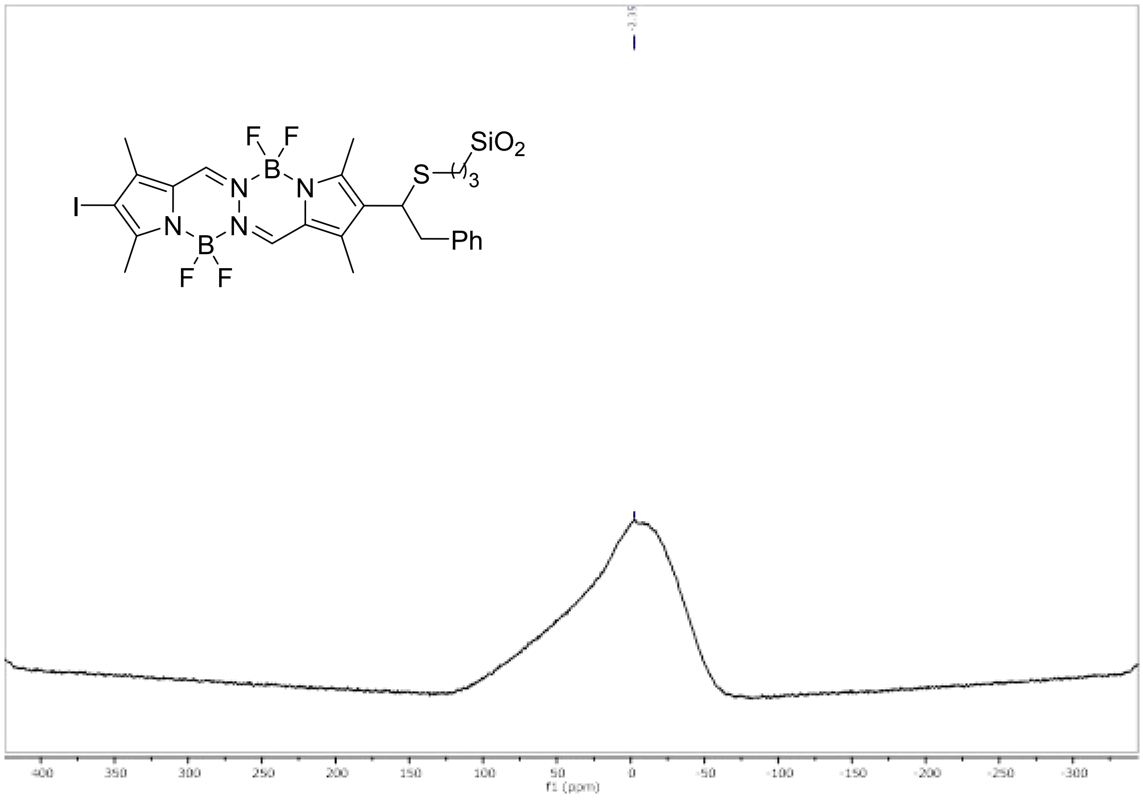

¹H NMR (400 MHz, $CDCl_3$)

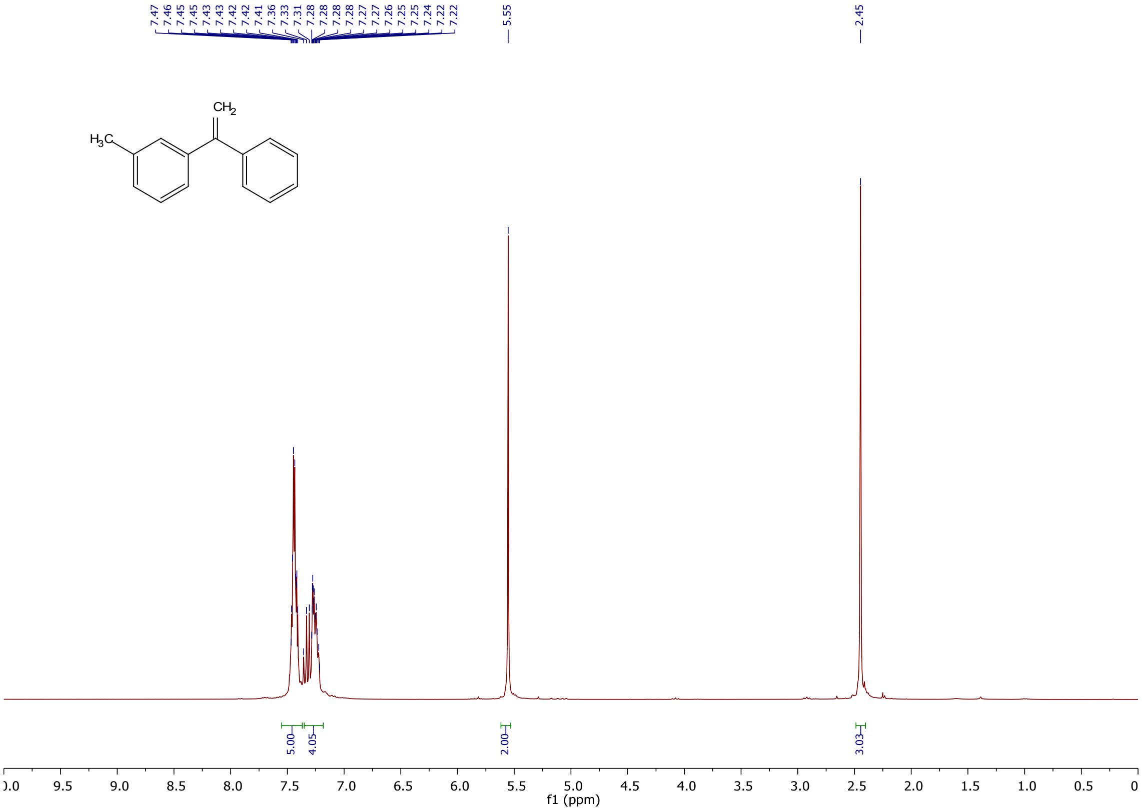

¹³C NMR (101 MHz, $CDCl_3$)

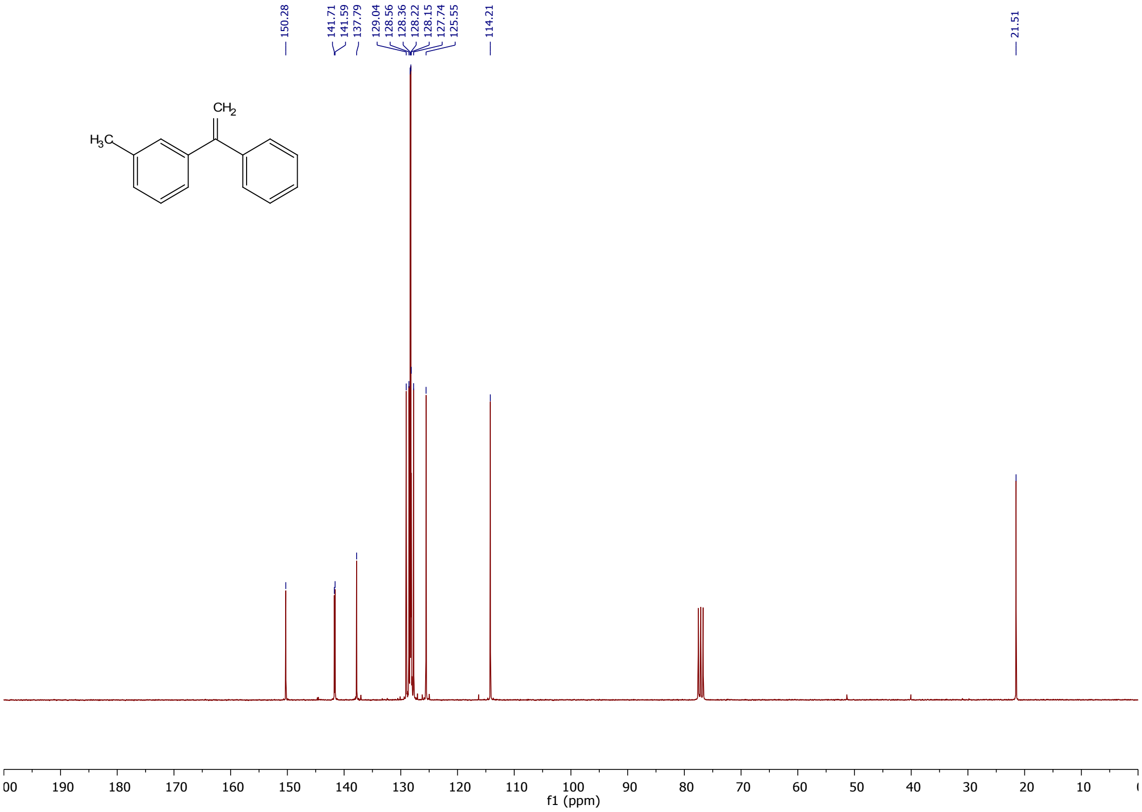

^{13}C-DEPT NMR (101 MHz, $CDCl_3$)

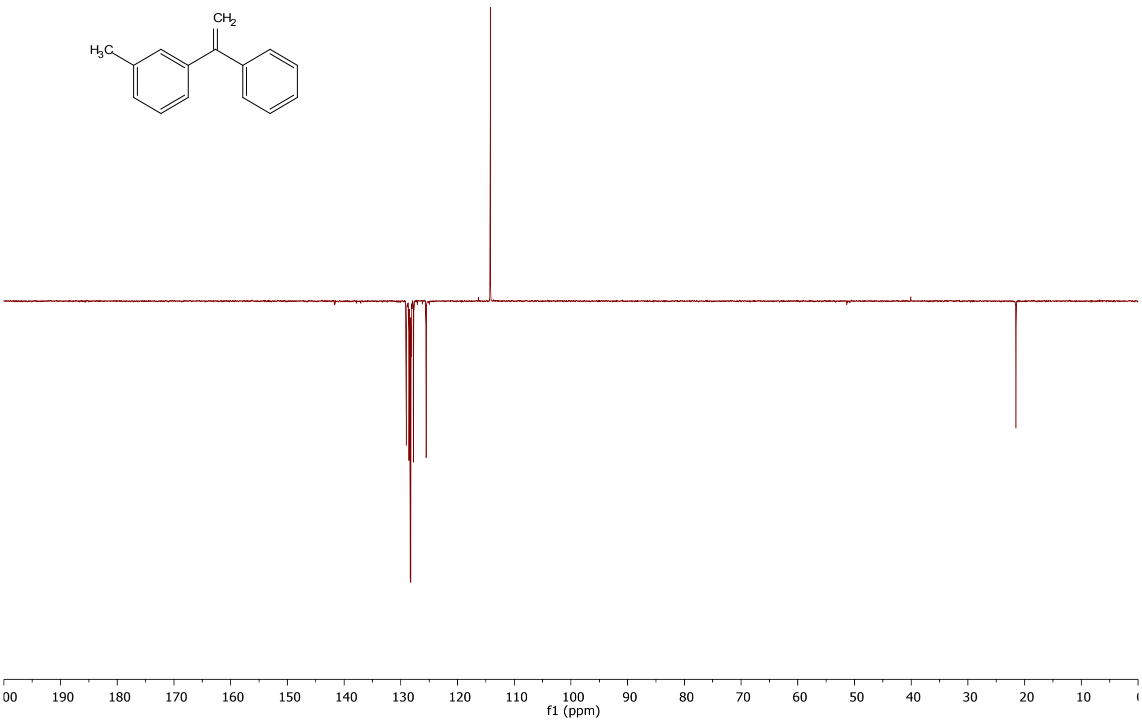

[1]**H NMR** (400 MHz, $CDCl_3$)

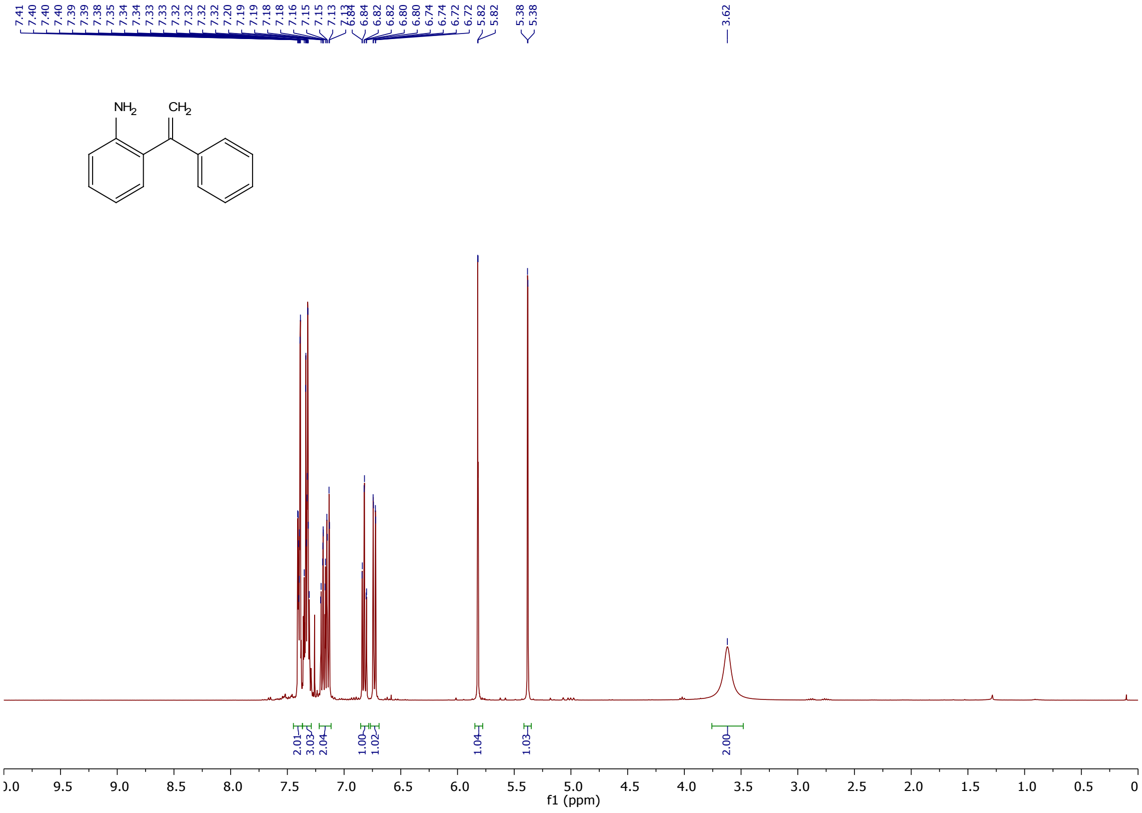

[13]**C NMR** (101 MHz, $CDCl_3$)

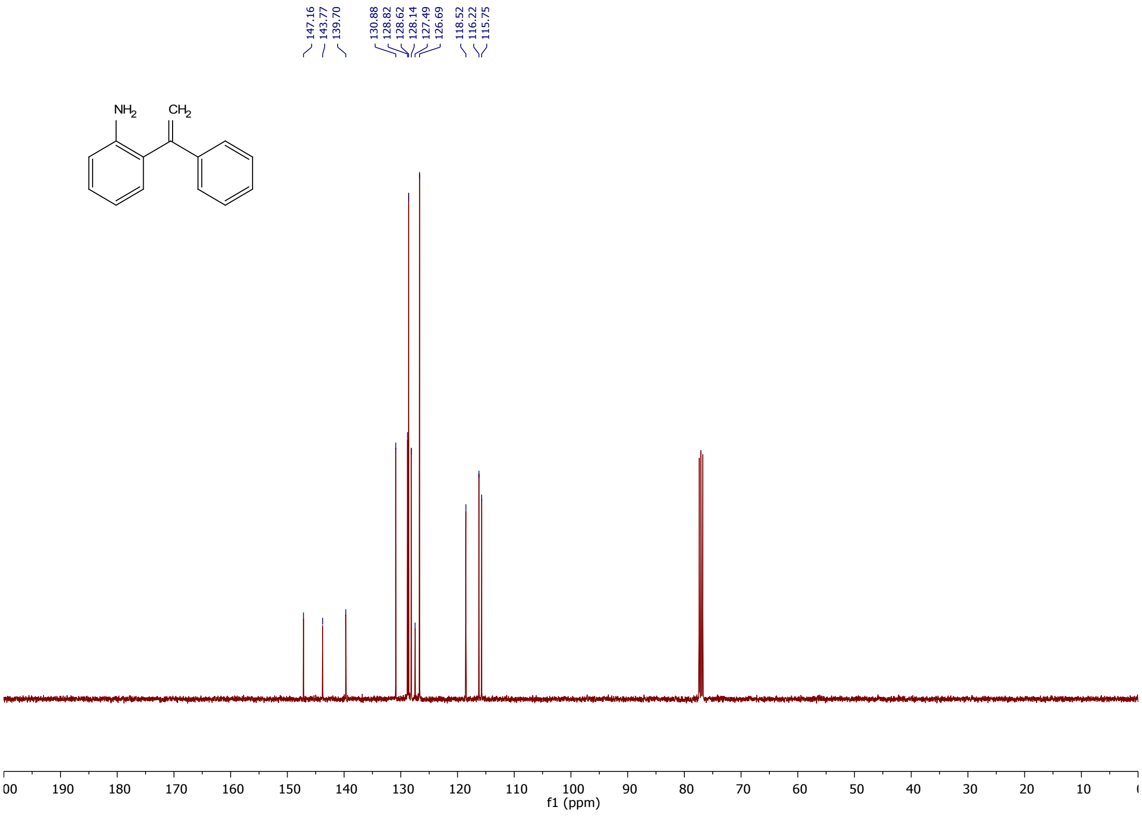

^{13}C-DEPT NMR (101 MHz, $CDCl_3$)

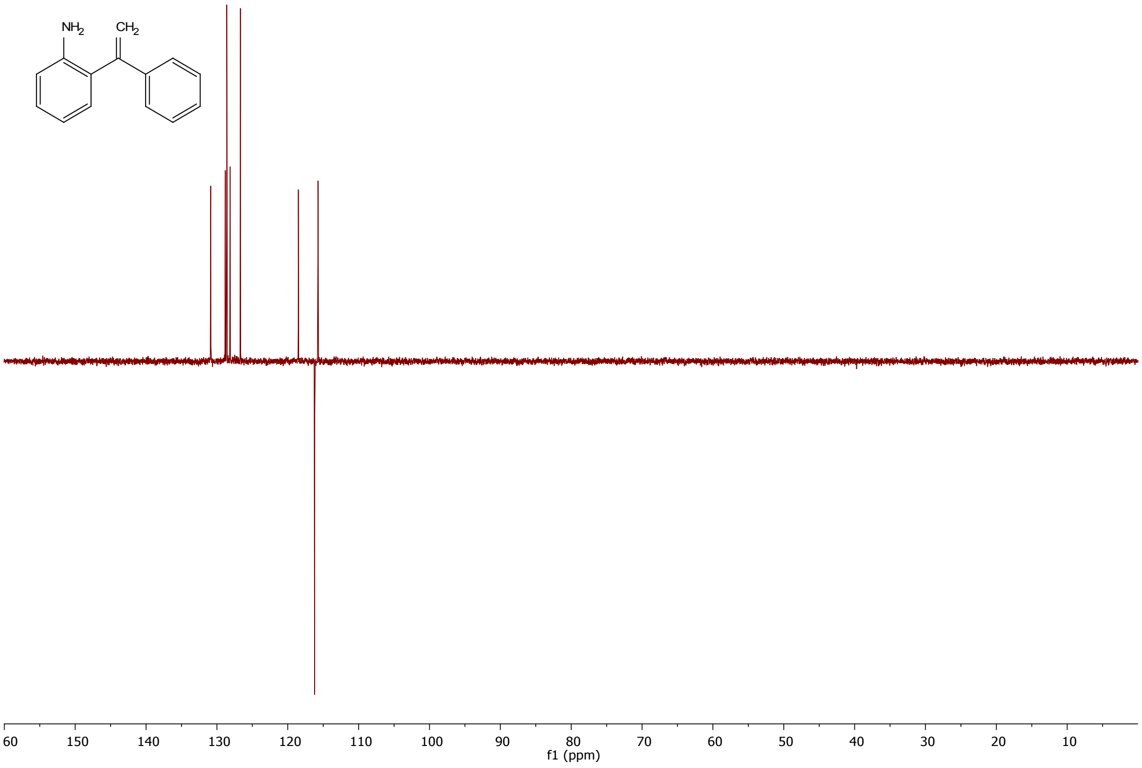

^{1}H NMR (400 MHz, CD_3CN)

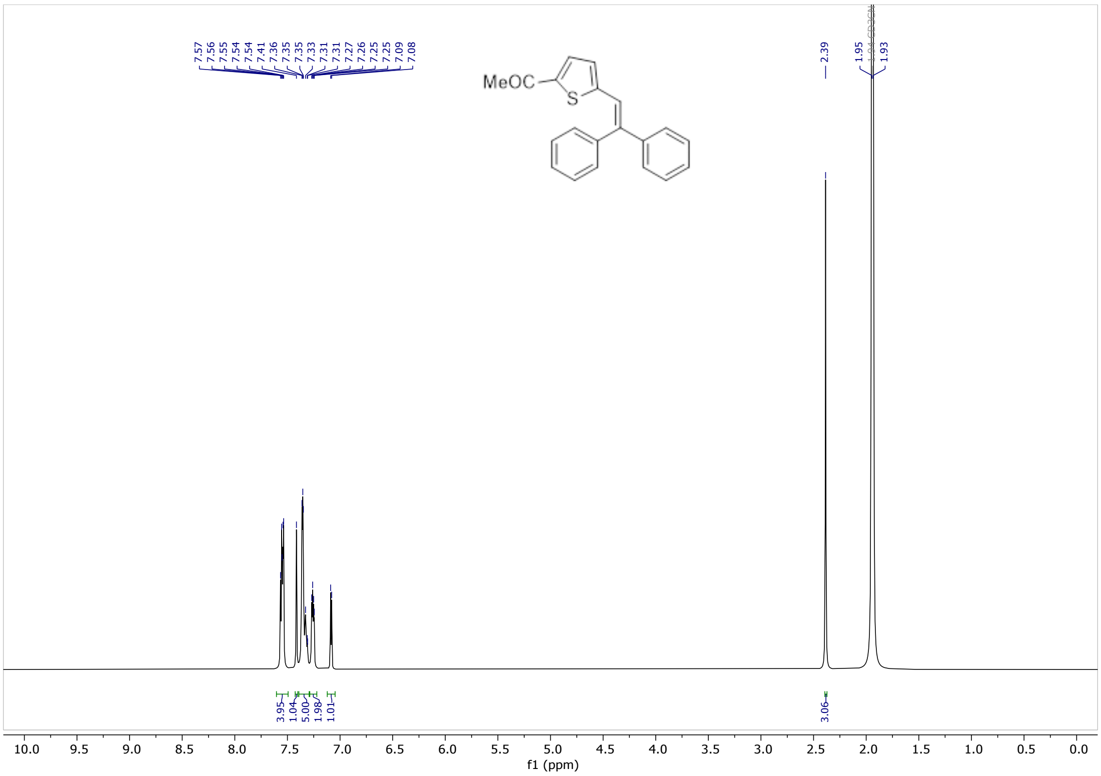

^{13}C NMR (101 MHz, CD_3CN)

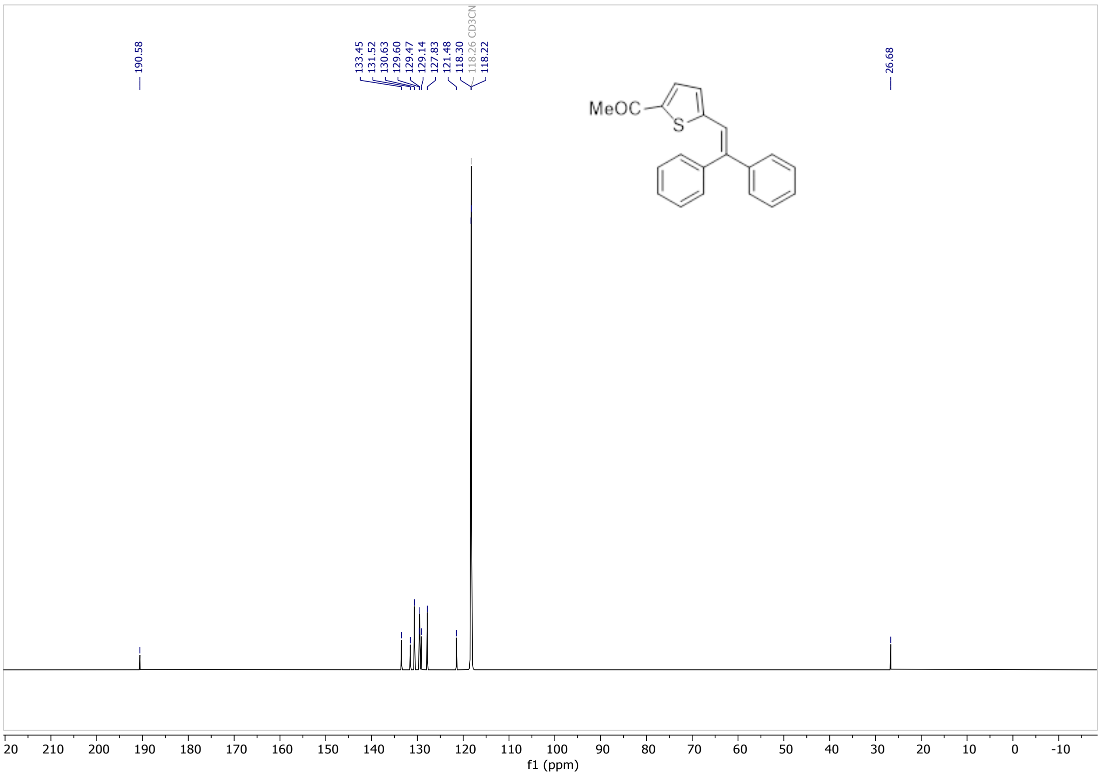

^{1}H NMR (400 MHz, CD_3CN)

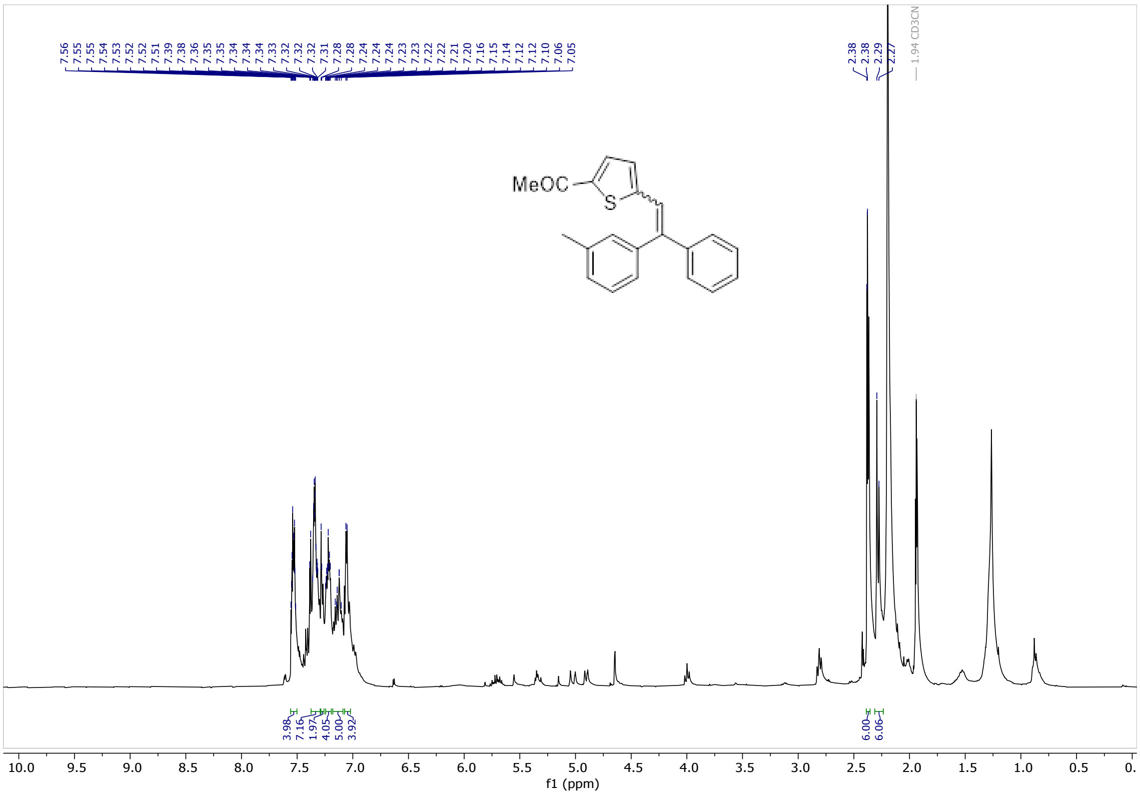

^{13}C NMR (101 MHz, CD_3CN)

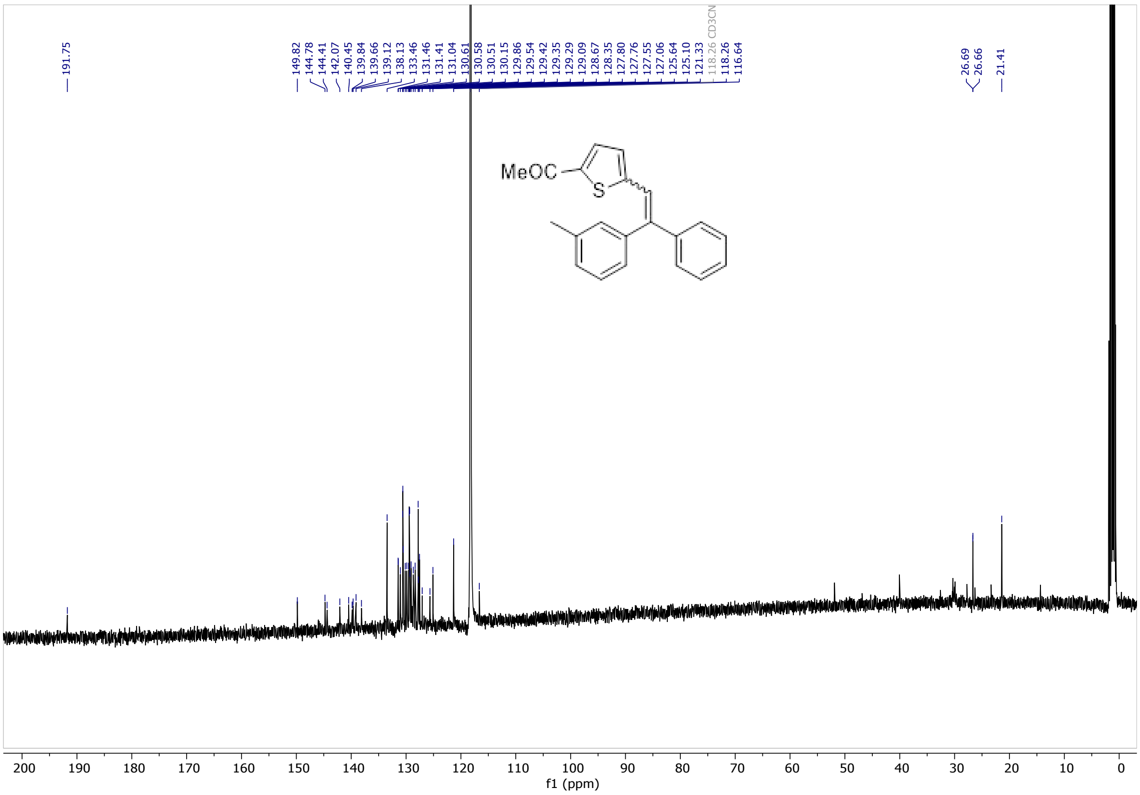

¹H NMR (400 MHz, CD_3CN)

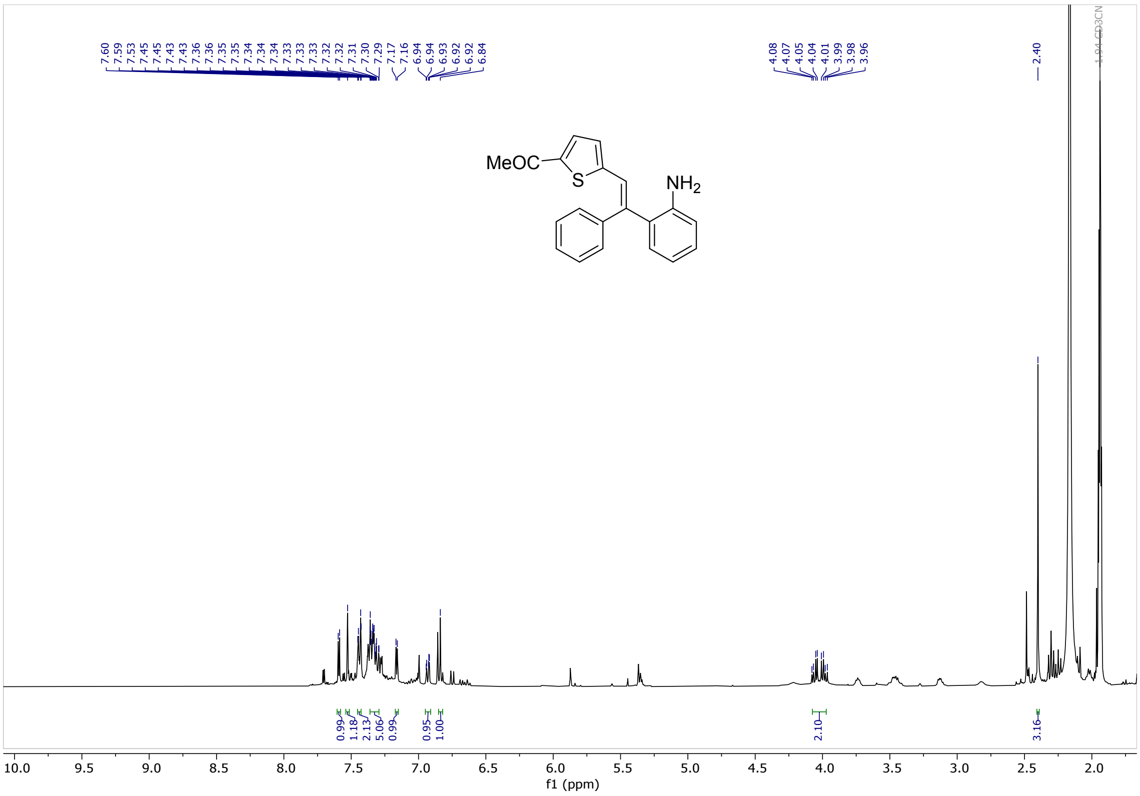

NOESY (400 MHz, CD_3CN)

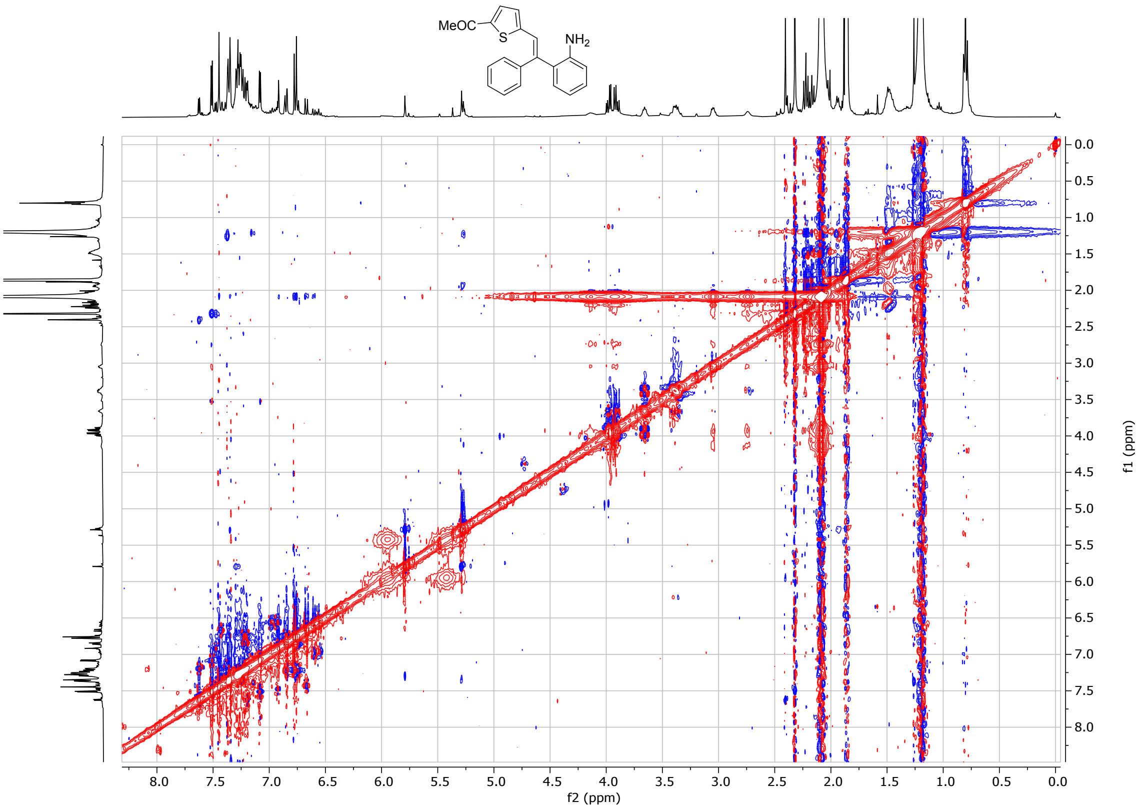

¹³C NMR (101 MHz, CD_3CN)

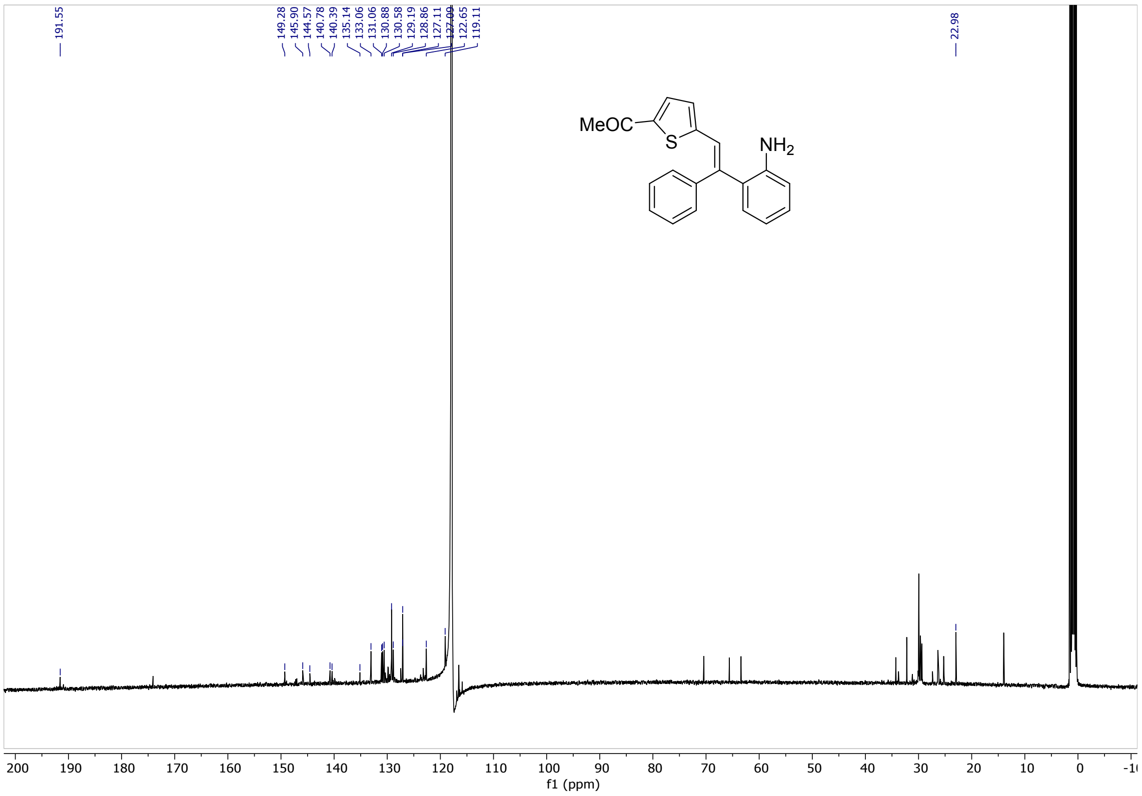

^{1}**H NMR** (400 MHz, CD_3CN)

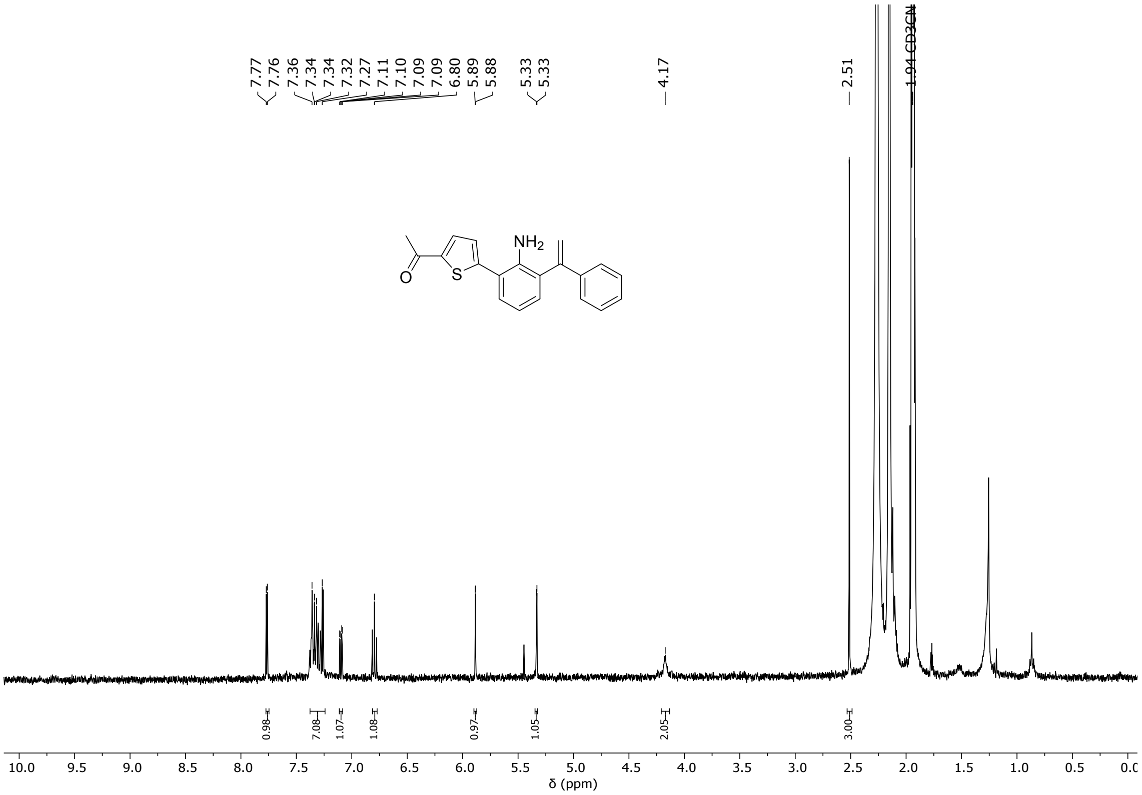

NOESY (400 MHz, CD_3CN)

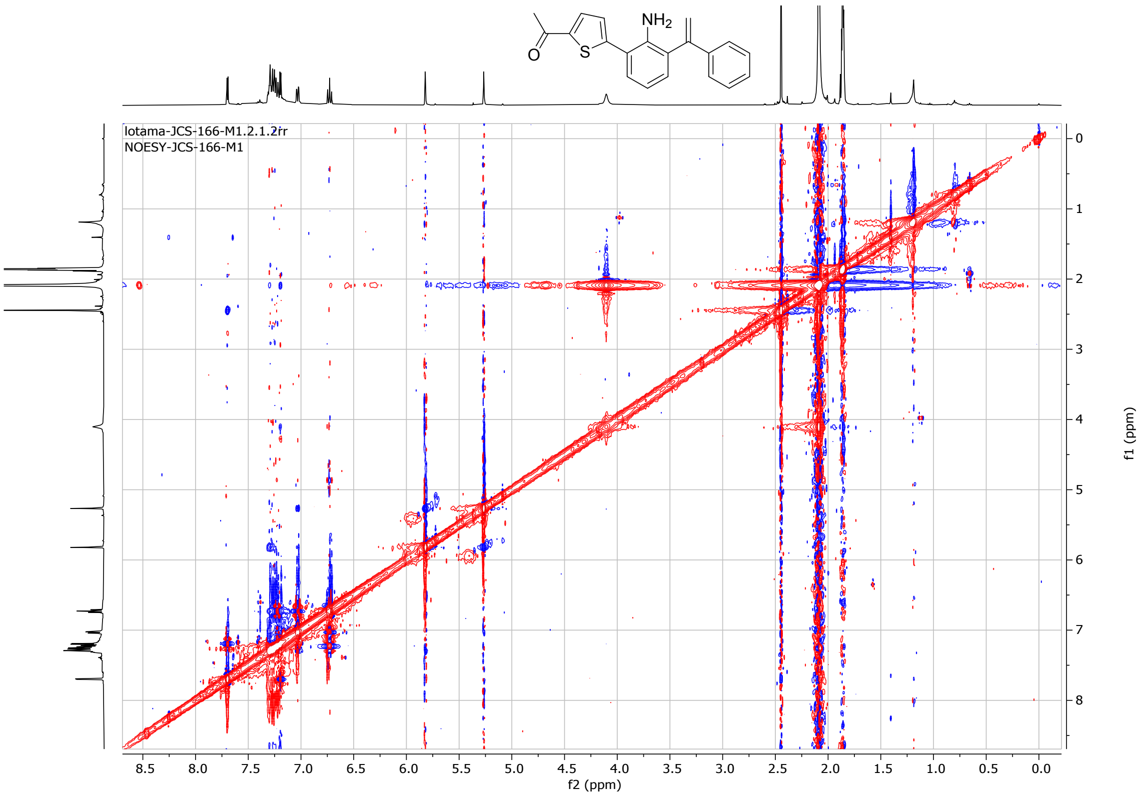

^{13}C NMR (101 MHz, CD_3CN)

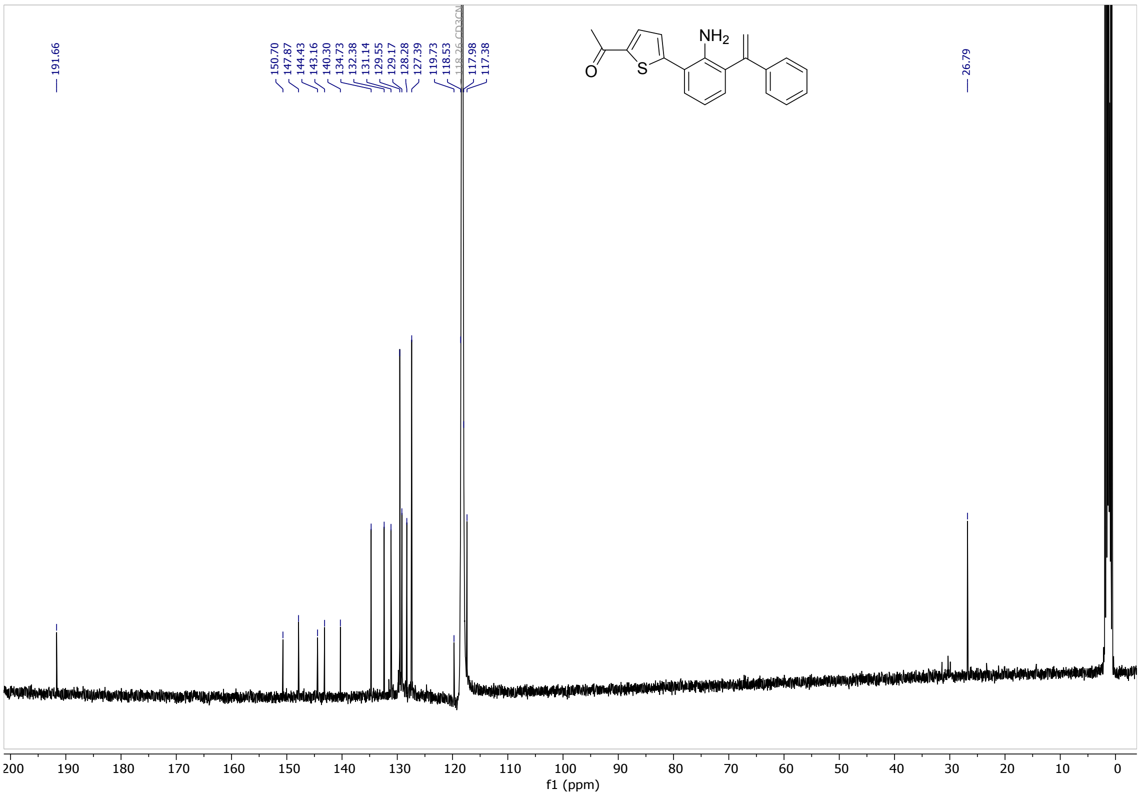

^{1}H NMR (400 MHz, $CDCl_3$)

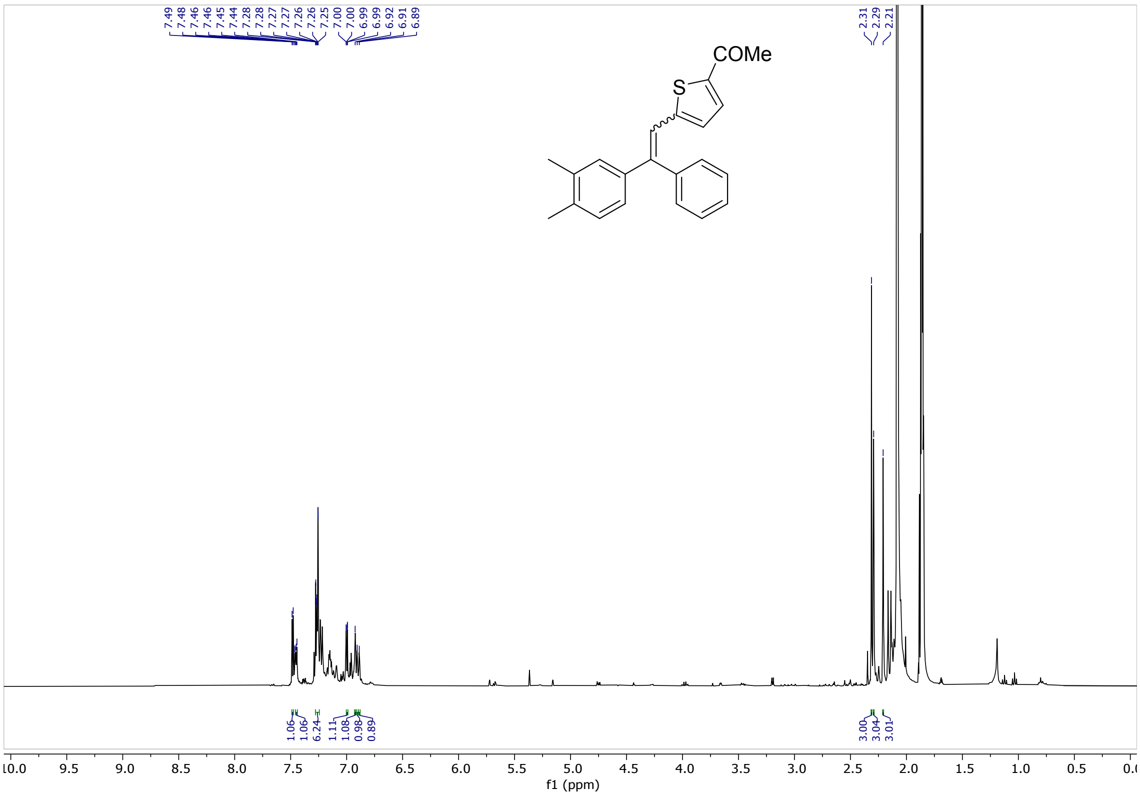

NOESY (400 MHz, $CDCl_3$)

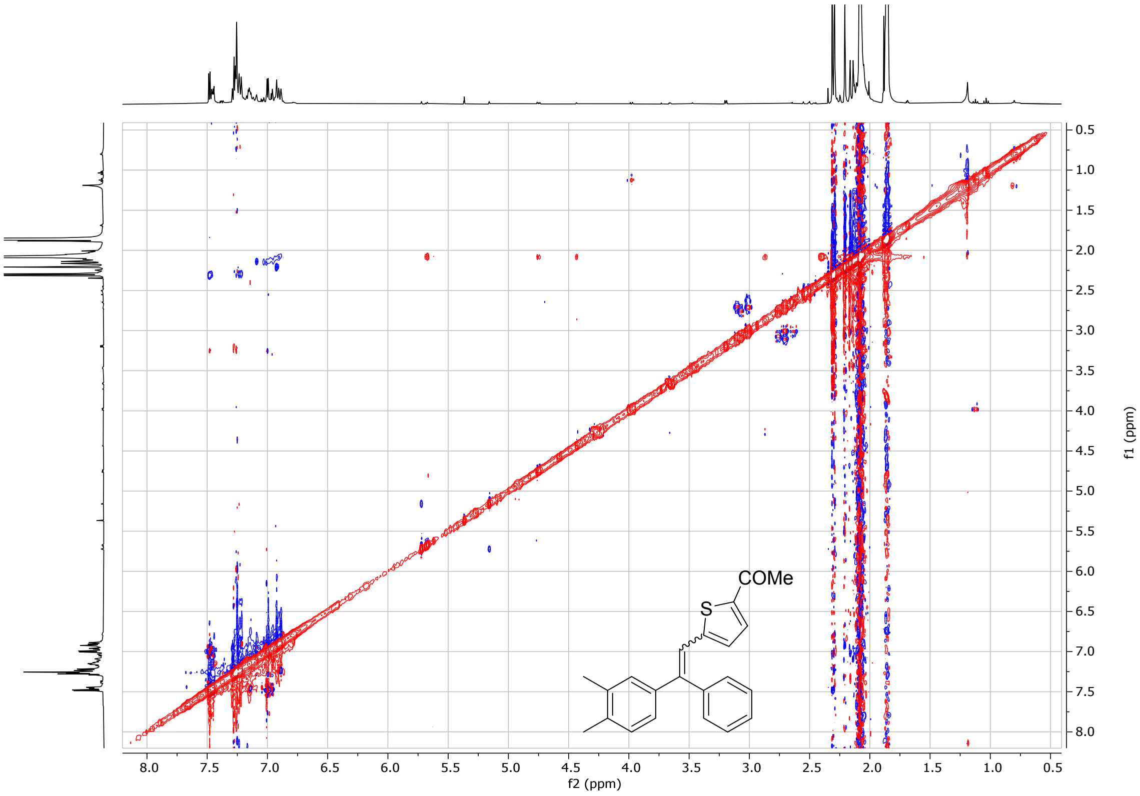

^{13}C NMR (101 MHz, $CDCl_3$)

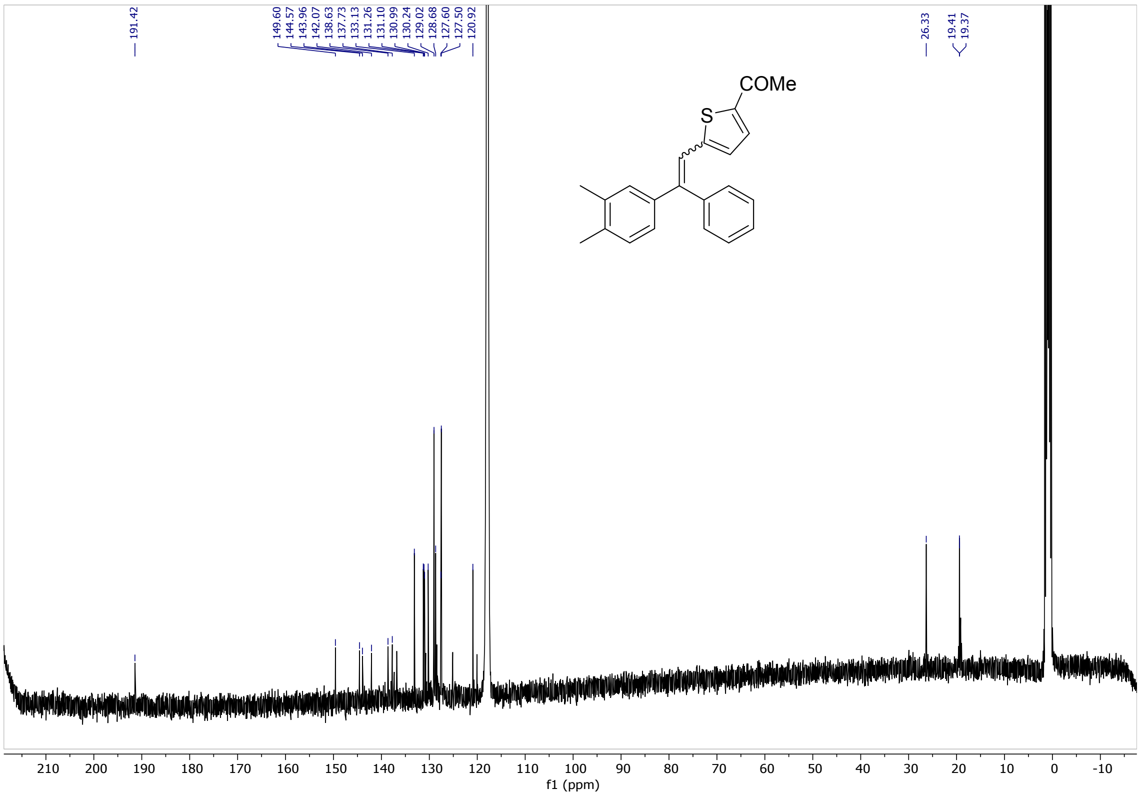

^{1}H NMR (400 MHz, Acetone)

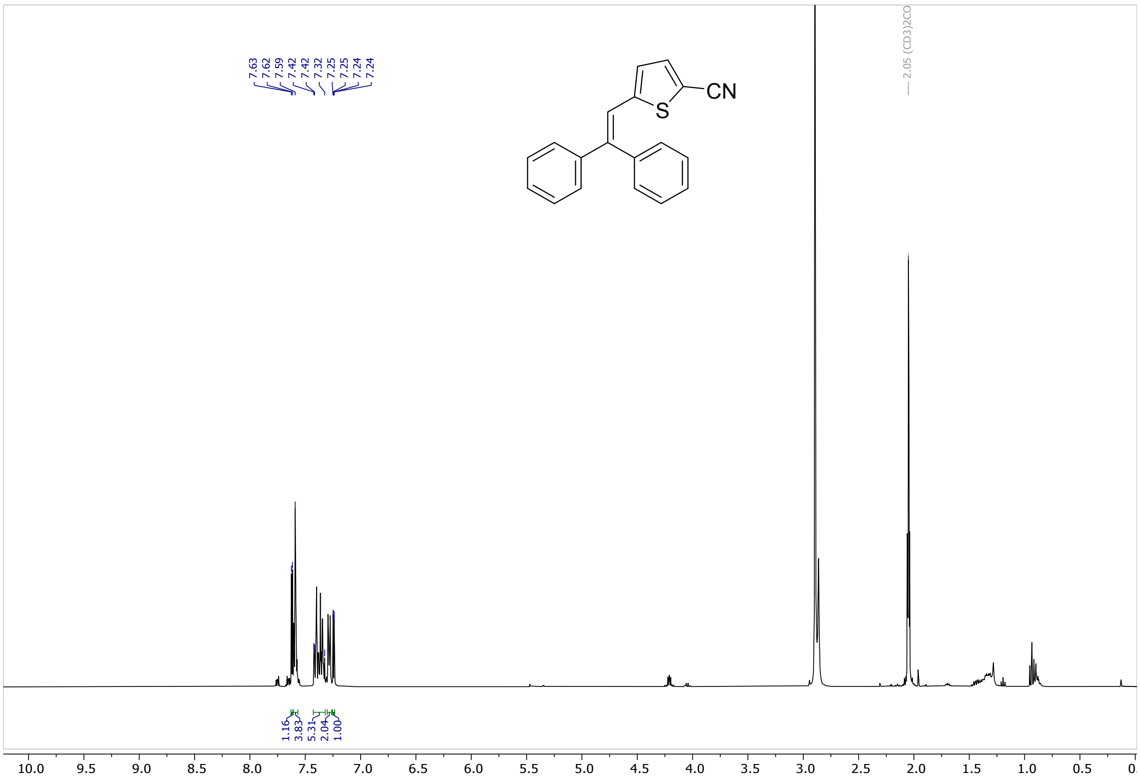

^{13}C NMR (101 MHz, Acetone)

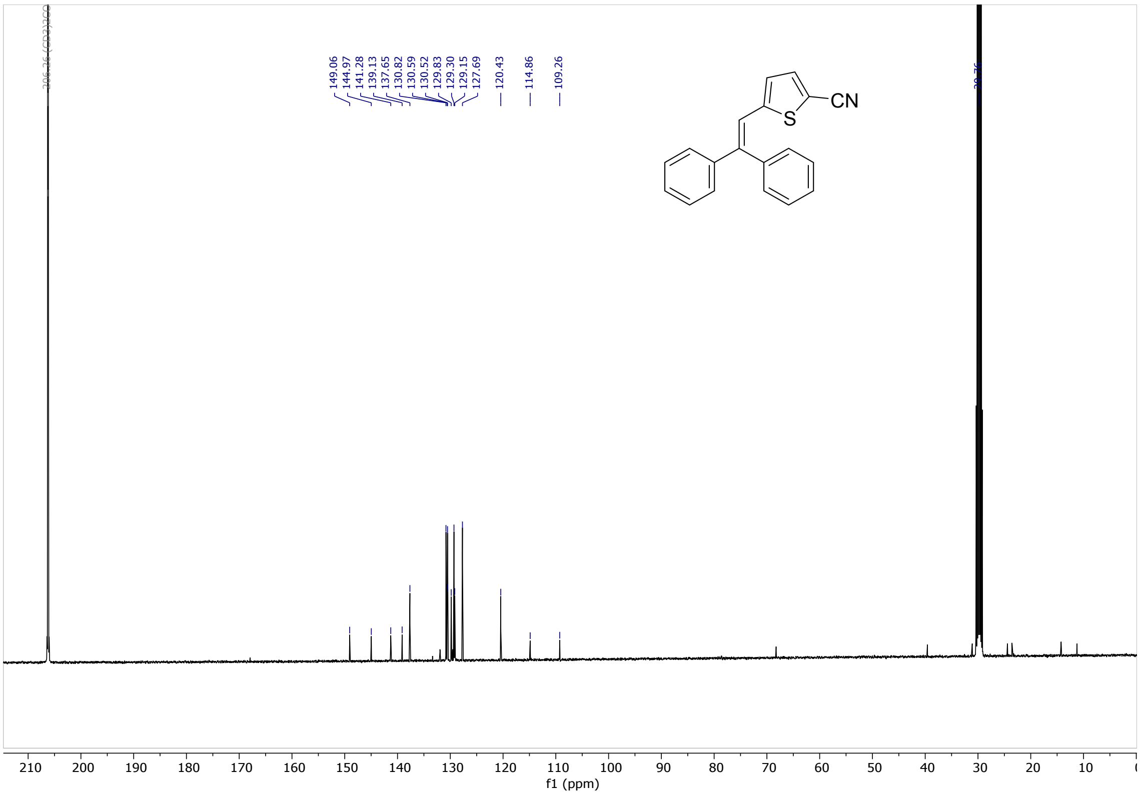

15- IR copies

IR – BOPHY-2

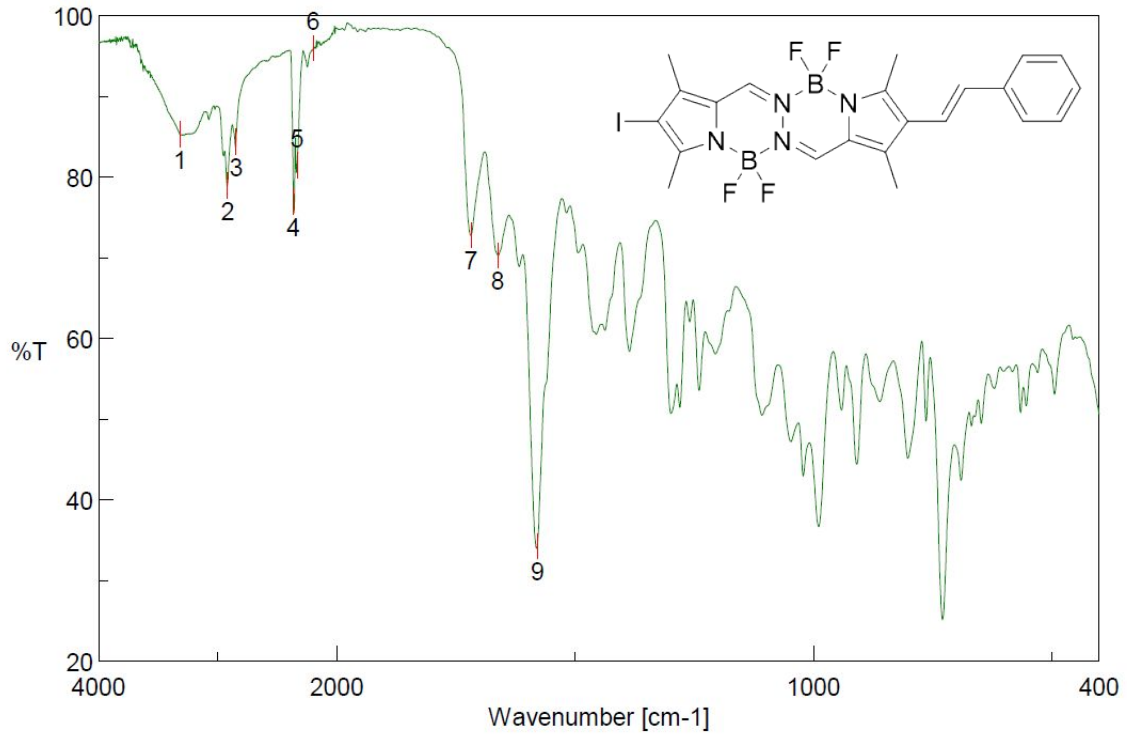

IR - SiO_2@BOPHY-2

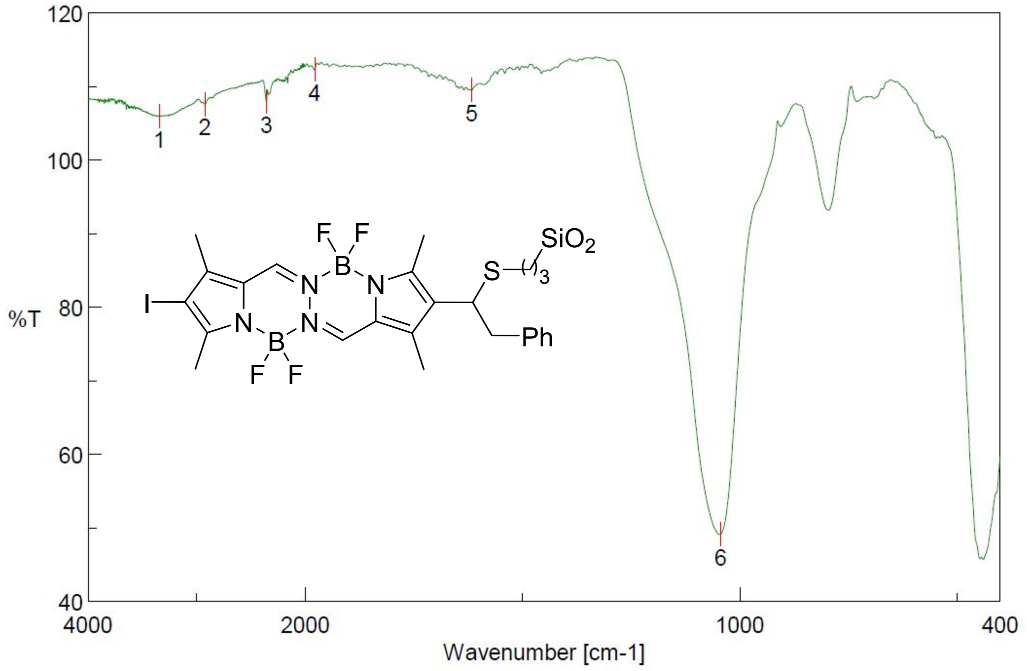

Supplement: Supplementary file 1 — pg3c00062_si_001.pdf [file pg3c00062_si_001.pdf]
